# Hyperactive mTORC1 in lung mesenchyme induces endothelial cell dysfunction and pulmonary vascular remodeling

**DOI:** 10.1172/JCI172116

**Published:** 2023-12-20

**Authors:** Susan M. Lin, Ryan Rue, Alexander R. Mukhitov, Akansha Goel, Maria C. Basil, Kseniya Obraztsova, Apoorva Babu, Slaven Crnkovic, Owen A. Ledwell, Laura T. Ferguson, Joseph D. Planer, Ana N. Nottingham, Kanth Swaroop Vanka, Carly J. Smith, Edward Cantu, Grazyna Kwapiszewska, Edward E. Morrisey, Jillian F. Evans, Vera P. Krymskaya

**Affiliations:** 1 Division of Pulmonary, Allergy, and Critical Care Medicine, Department of Medicine, Perelman School of Medicine,; 2 Lung Biology Institute, and; 3 Penn Cardiovascular Institute, University of Pennsylvania, Philadelphia, Pennsylvania, USA.; 4 Division of Physiology, Medical University of Graz, Graz, Austria.; 5 Ludwig Boltzmann Institute for Lung Vascular Research, Graz, Austria.; 6 Institute for Lung Health, Justus-Liebig University Giessen, Giessen, Germany.; 7 Department of Surgery, Perelman School of Medicine, University of Pennsylvania, Philadelphia, Pennsylvania, USA.

**Keywords:** Cell Biology, Vascular Biology, Endothelial cells, Mouse models

## Abstract

Lymphangioleiomyomatosis (LAM) is a progressive cystic lung disease caused by tuberous sclerosis complex 1/2 (*TSC1/2*) gene mutations in pulmonary mesenchymal cells, resulting in activation of the mechanistic target of rapamycin complex 1 (mTORC1). A subset of patients with LAM develop pulmonary vascular remodeling and pulmonary hypertension. Little, however, is known regarding how LAM cells communicate with endothelial cells (ECs) to trigger vascular remodeling. In end-stage LAM lung explants, we identified EC dysfunction characterized by increased EC proliferation and migration, defective angiogenesis, and dysmorphic endothelial tube network formation. To model LAM disease, we used an mTORC1 gain-of-function mouse model with a *Tsc2* KO (*Tsc2^KO^*) specific to lung mesenchyme (*Tbx4^LME-Cre^* *Tsc2^fl/fl^*), similar to the mesenchyme-specific genetic alterations seen in human disease. As early as 8 weeks of age, ECs from mice exhibited marked transcriptomic changes despite an absence of morphological changes to the distal lung microvasculature. In contrast, 1-year-old *Tbx4^LME-Cre^* *Tsc2^fl/fl^* mice spontaneously developed pulmonary vascular remodeling with increased medial thickness. Single-cell RNA-Seq of 1-year-old mouse lung cells identified paracrine ligands originating from *Tsc2^KO^* mesenchyme, which can signal through receptors in arterial ECs. These ECs had transcriptionally altered genes including those in pathways associated with blood vessel remodeling. The proposed pathophysiologic mesenchymal ligand–EC receptor crosstalk highlights the importance of an altered mesenchymal cell/EC axis in LAM and other hyperactive mTORC1–driven diseases. Since ECs in patients with LAM and in *Tbx4^LME-Cre^* *Tsc2^fl/fl^* mice did not harbor *TSC2* mutations, our study demonstrates that constitutively active mTORC1 lung mesenchymal cells orchestrated dysfunctional EC responses that contributed to pulmonary vascular remodeling.

## Abstract 2

<p>Hyperactive mTORC1-driven mesenchymal cells can act as a signaling hub that orchestrates dysregulated cellular responses and leads to pulmonary vascular remodeling.</p>

## Introduction

Lymphangioleiomyomatosis (LAM) is a destructive lung disease characterized by diffuse parenchymal cystic airspace enlargement, airflow obstruction, and chylothorax from lymphatic involvement (1–6). In addition, some patients with LAM develop pulmonary vascular remodeling with intimal and medial hypertrophy of pulmonary arteries (7) and subsequent pulmonary hypertension (7–9). There are 2 forms of LAM: sporadic and inherited. Both sporadic and inherited LAM result from loss-of-function mutations in the tuberous sclerosis complex 1/2 (*TSC1/TSC2*) genes, resulting in constitutive activation of the mechanistic target of rapamycin (mTOR) pathway, specifically mTOR complex 1 (mTORC1) (10–12). This genetic mutation only occurs in a distinct population of mesenchymal cells known as LAM cells (13). LAM cells, which are estimated to be less than 5% of the total lung cell population, are localized in the pulmonary parenchyma adjacent to cystic lesions, bronchioles, vasculature, and lymphatics (7). Pulmonary lesions formed by LAM cells consist of mixed mesenchymal cell populations with myofibroblast-like, spindle-shaped cells expressing smooth muscle–specific markers and epithelioid-like cells expressing the glycoprotein gp100 (5).

Single-cell RNA-Seq (scRNA-Seq) of LAM lungs has shown marked transcriptomic changes in cell populations surrounding LAM cell lesions, including mesenchymal and epithelial cells (14). The effect of aberrant mTOR signaling in LAM cells on endothelial cells (ECs) and pulmonary vasculature has not been previously investigated. Thus, it is unknown how LAM cells, localized in the lung interstitium, alter the behavior and function of vascular ECs. The role of mTOR hyperactivation on the LAM mesenchymal cell/EC axis is particularly significant, as mTOR pathway activation has been recently implicated in the progression of pulmonary vascular remodeling in pulmonary hypertension (PH) (15–19). Prior research has demonstrated that mTORC2-dependent activation of mTORC1 through oxidative stress is a major cause of vascular remodeling by coordinating pulmonary artery smooth muscle cell (PASMC) metabolism and proliferation (15). Follow-up studies have identified mTORC1 as a mediator of PASMC function under hypoxic conditions (20) and in pathogenic right ventricular (RV) remodeling (21). A more recent study showed that in smooth muscle cell–specific conditional KOs of *Raptor* or *Rictor* in murine models, inhibition of mTORC1 attenuates the development of PH, whereas inhibition of mTORC2 causes spontaneous PH (19).

To better understand the role of mTORC1 activation in mesenchymal cells on lung EC dysfunction and pulmonary vascular remodeling, we generated primary cultures of ECs from patients with LAM and demonstrated that dysregulated WNT signaling in mTORC1 hyperactive mesenchymal cells contributed to EC dysfunction. To further investigate how mesenchymal cells alter EC behavior, we generated a murine model with selective *Tsc2* deletion in pulmonary mesenchymal progenitors (*Tbx4^LME-Cre^ Tsc2^fl/fl^*) (14). We previously found that *Tbx4^LME-Cre^ Tsc2^fl/fl^* mice with mesenchyme-specific loss of *Tsc2* develop an age- and sex-dependent decline in pulmonary function and destruction of alveolar lung structure (14). In this study, we focused on the effects of the mutated lung mesenchyme on pulmonary ECs and found changes in EC function and the transcriptomic signature. Moreover, parallel to the development of PH in a subset of patients with late-stage LAM, aged *Tbx4^LME-Cre^ Tsc2^fl/fl^* mice spontaneously developed pulmonary vascular remodeling with concurrent elevation in RV systolic pressure (RVSP). Taken together, our research demonstrates that mTORC1 hyperactivation in lung mesenchyme caused vascular EC dysfunction through proliferation and phenotypic transformation of pulmonary artery ECs. This aberrant signaling response subsequently resulted in pulmonary vascular remodeling and further emphasizes the pathogenic importance of the mesenchymal cell/EC communication axis as a potential cause of pulmonary vascular remodeling.

## Results

### ECs from LAM lung explants are characterized by a hyperproliferative phenotype with reduced angiogenic capacity in a 2D monoculture.

**Figure 1:**
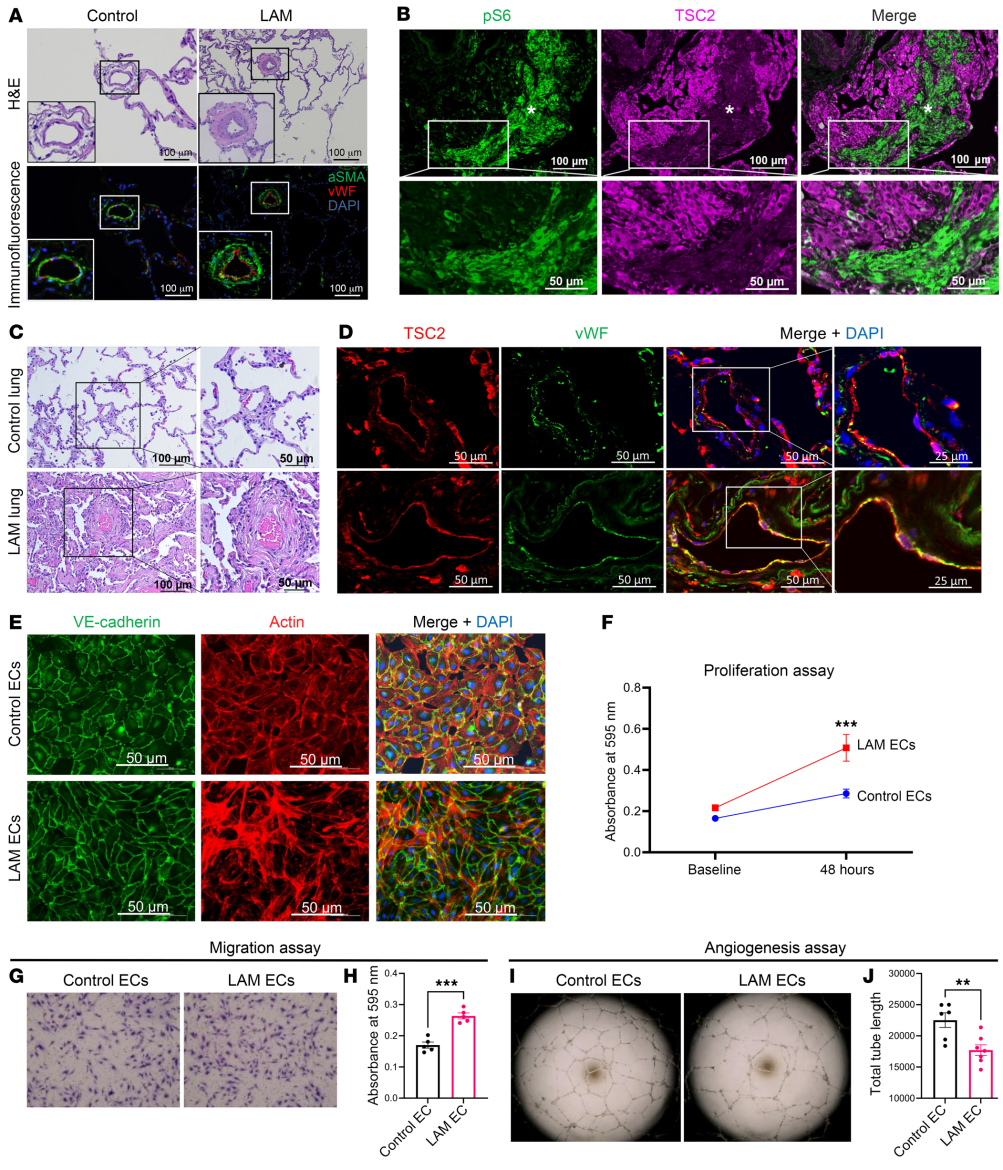
Characterization of pulmonary ECs from patients with LAM. Representative images of H&E- and immunofluorescence-stained LAM lung (*n* = 3) compared with age- and sex-matched control human lung (*n* = 3) showing (**A**) remodeled distal vasculature detected with antibodies against vWF (red) and αSMA (green) and (**B**) increased intimal fibrosis and medial thickening by H&E staining. DAPI was used to detect nuclei (blue). Scale bars: 50 μm and 100 μm. Insets: Original magnification, ×2. (**C**) Representative images of dual immunofluorescence staining of human LAM lung (*n* = 3) demonstrating loss of TSC2 (tuberin, magenta) expression in a LAM lesion and concurrent activation of pS6 (marker of mTOR activation, green) and DAPI (nuclei, blue). White asterisks show LAM micronodules. Scale bars: 50 μm and 100 μm. (**D**) Representative images of positive immunoreactivity for TSC2 protein (tuberin, magenta) and vWF (green) in ECs lining the pulmonary vasculature in both human control (*n* = 4) and LAM lungs (*n* = 4). DAPI (nuclei, blue). Scale bars: 25 μm and 50 μm. (**E**) LAM and control pulmonary ECs labeled with antibodies against endothelial marker vWF (green) and F-actin (rhodamine phalloidin; red). Nuclei were counterstained with DAPI (blue). Scale bars: 50 μm. (**F**) Proliferation of control and LAM ECs over 48 hours using a crystal violet growth assay. (**G**) Representative images of migration of control (*n* = 5) and LAM (*n* = 5) ECs following a Boyden chamber assay. (**H**) Statistical analysis of EC migration quantified 24 hours after migration using 2-tailed *t* tests of crystal violet absorbence. (**I**) Representative image of in vitro angiogenesis in 2D of control (*n* = 6) and LAM (*n* = 6) ECs. (**J**) Total tube lengths at 24 hours of growth on Matrigel were analyzed using the Angiogenesis Analyzer plugin in ImageJ (NIH). Data are presented as the mean ± SEM. **P < 0.01 and ***P < 0.001, by 2-tailed *t* test.

To elucidate the possibility of vascular EC dysfunction in LAM, we performed histopathological analysis of diseased lung explants obtained from LAM patients with LAM at the time of lung transplantation. End-stage LAM lung explants exhibit diffuse parenchymal cysts (not shown; for an overview of LAM as a cystic lung disease, see ref. 22), lymphatic involvement, as well as remodeled vessels (Figure 1A) with concentric fibrosis of the intima and media (Figure 1B). Interspersed in the lung parenchyma are LAM micronodules consisting of mesenchymal LAM cells, which stain positive for phosphorylated ribosomal protein S6 (pS6), a marker of mTORC1 activation (12) (Figure 1C). LAM cells of mesenchymal origin are the only cell types in the LAM lung to harbor *TSC2* mutations, whereas pulmonary cells of other lineages, including ECs, do not harbor *TSC2* mutations. As such, ECs from both control and LAM lungs had TSC2 expression detected by dual immunohistochemistry (Figure 1D) and RNAscope (Supplemental Figure 1A; supplemental material available online with this article; https://doi.org/10.1172/JCI172116DS1).

To purify and characterize ECs, LAM lung specimens with distal pulmonary vasculature were mechanically dissected and enzymatically dissociated into single-cell suspensions as previously described (23, 24). To ensure capture of distal cells, we used only peripheral lung segments, identified by the presence of visceral pleura. Microdissection was performed along the bronchovascular bundle, and vascular ECs were isolated using antibody magnetic bead sorting and grown to confluence on fibronectin-coated plates (Supplemental Figure 1B). To confirm cell-type identity and purity, we examined expression patterns by flow cytometry with canonical lineage markers (CD45 for immune cell populations, EPCAM for epithelial cell populations, and CD31 for EC populations; Supplemental Figure 1C). An immunoblot of primary fibroblasts derived from LAM lung was notable for a significant decrease in TSC2 expression (Supplemental Figure 1D), while there were no significant differences in TSC2 protein expression in purified ECs isolated from LAM lungs compared with control lungs (Supplemental Figure 1E). Control pulmonary vascular ECs exhibited a polygonal morphology with an organized cobblestone appearance. Compared with age- and sex-matched controls, LAM-derived ECs were dysmorphic, hypertrophic, and less uniform in appearance, with increased randomly oriented actin fibers (Figure 1E).

Similar to existing studies of ECs isolated from remodeled PH vasculature (25, 26), we found that LAM ECs were more proliferative (Figure 1F) with increased migration (Figure 1, G and H), but had a reduced angiogenic capacity as measured by an in vitro tube formation assay (Figure 1I). On these 2D monocultures, LAM ECs exhibited decreased tube lengths compared with control ECs, indicating reduced angiogenic potential when grown in the absence of support cells (Figure 1J).

### LAM lung fibroblasts increase the angiogenic potential of LAM ECs.

**Figure 2:**
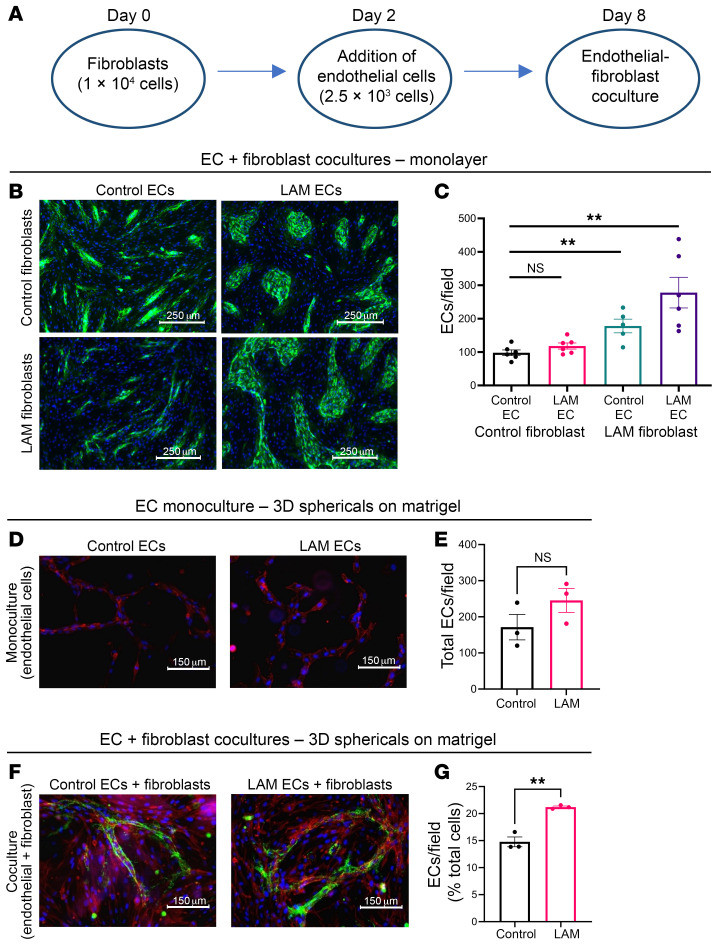
Increased EC proliferation and in vitro vasculogenesis in 3D EC-fibroblast coculture systems. (**A**) Schematic for EC-fibroblast coculture experiments. (**B**) Representative images of EC-fibroblast cocultures in a monolayer at day 8 using primary cells isolated from LAM lung explants and age-matched control human lung. ECs were stained with vWF (green) and nuclei were counterstained with DAPI (blue). Scale bars: 250 μm. Original magnification, ×10 and ×20. (**C**) Quantification of the number of ECs per field in the EC-fibroblast cocultures. (**D**) Representative images of 3D control (*n* = 3) and LAM (*n* = 3) EC sphericals in serum-free Matrigel matrix. Cells were stained with rhodamine phalloidin (red) to visualize EC structures. Scale bars: 150 μm. (**E**) Quantification of the total number of ECs per field by nonparametric Kruskal-Wallis ANOVA with Siegel (Bonferroni’s) correction. (**F**) Representative images of 3D sphericals with control (*n* = 3) and LAM (*n* = 3) ECs grown in the presence of fibroblasts from control human and LAM lung, respectively, on serum-free Matrigel matrix. ECs were immunostained with vWF (green), and nuclei were counterstained with DAPI (blue). (**G**) Quantification of ECs per field (percentage of total cells) in 3D cocultures. **P < 0.01, by nonparametric Kruskal-Wallis ANOVA test with Siegel (Bonferroni’s) correction for post hoc, pairwise contrasts (**C**, **E**, and **G**).

As vascular ECs from LAM lungs do not harbor *TSC1* or *TSC2* mutations and do not have any previously known roles in LAM pathogenesis, it is unclear how mesenchymal LAM cells with constitutive mTORC1 hyperactivation alter vascular EC function. We speculated that this could occur through paracrine signaling between mesenchymal and vascular ECs. Thus, to assess EC-mesenchymal crosstalk through cell-cell and cell-matrix interactions, primary ECs and fibroblasts from patients with LAM or control human lung were cocultured in 2D on fibronectin-coated 12-well plates (Figure 2A). In 2D cocultures with LAM fibroblasts, ECs from LAM lung demonstrated a very different morphology, with increased affinity for self-segregation and self-organization (Figure 2B). To investigate how EC behavior is altered by surrounding cells and the pulmonary microenvironment, we developed EC-fibroblast cocultures to interrogate cell-cell interactions and 3D EC-fibroblast cocultures to investigate cell-matrix interactions. First, ECs from LAM lungs were more proliferative than control ECs when cocultured with control fibroblasts, which increased further when ECs from LAM lung were cocultured with LAM fibroblasts (Figure 2C). Thus, LAM fibroblasts appear to have an additive effect on LAM EC growth and proliferation. Next, we assessed the ability of ECs from LAM lung to self-assemble and form endothelial tubes within 3D extracellular matrix–based (ECM-based) scaffolds (Figure 2D). When ECs were grown as a monoculture in 3D sphericals, we observed a trend toward increased EC proliferation, but a nonsignificant difference in total tube length and cell count between ECs from LAM lung and control human ECs (Figure 2E). However, when grown in coculture with LAM lung–derived fibroblasts, ECs from LAM lung developed more extensive tube networks (Figure 2F) with increased EC proliferation (Figure 2G). Taken together, the major changes in EC organization in 2D cocultures and the significant increase in cell count and tube formation in 3D cocultures suggest that mesenchymal cells from LAM lung induced EC proliferation and in vitro vasculogenesis.

### LAM scRNA-Seq reveals transcriptomic alterations in pulmonary vascular ECs.

To assess the gene expression profile in LAM ECs, we analyzed LAM scRNA-Seq data on LAM lung mesenchymal and EC clusters. Epithelial cells, immune cells, mesenchymal cells, and ECs were identified using canonical markers (27–30). ECs were subsequently subclassified for further analysis on the basis of EC-selective markers (27, 31). Pulmonary artery ECs were characterized by high expression of *GJA5* and *DKK2* genes; venous ECs (VECs) by *ACKR1* and *HDAC9*; lymphatic ECs by *PROX1*, capillary (CAP) ECs by *ILJR* (CAP1) or *EDNRB* (CAP2); and Car4^hi^ (Car4^+^) ECs by *CA4* and *CD34*.

**Figure 3:**
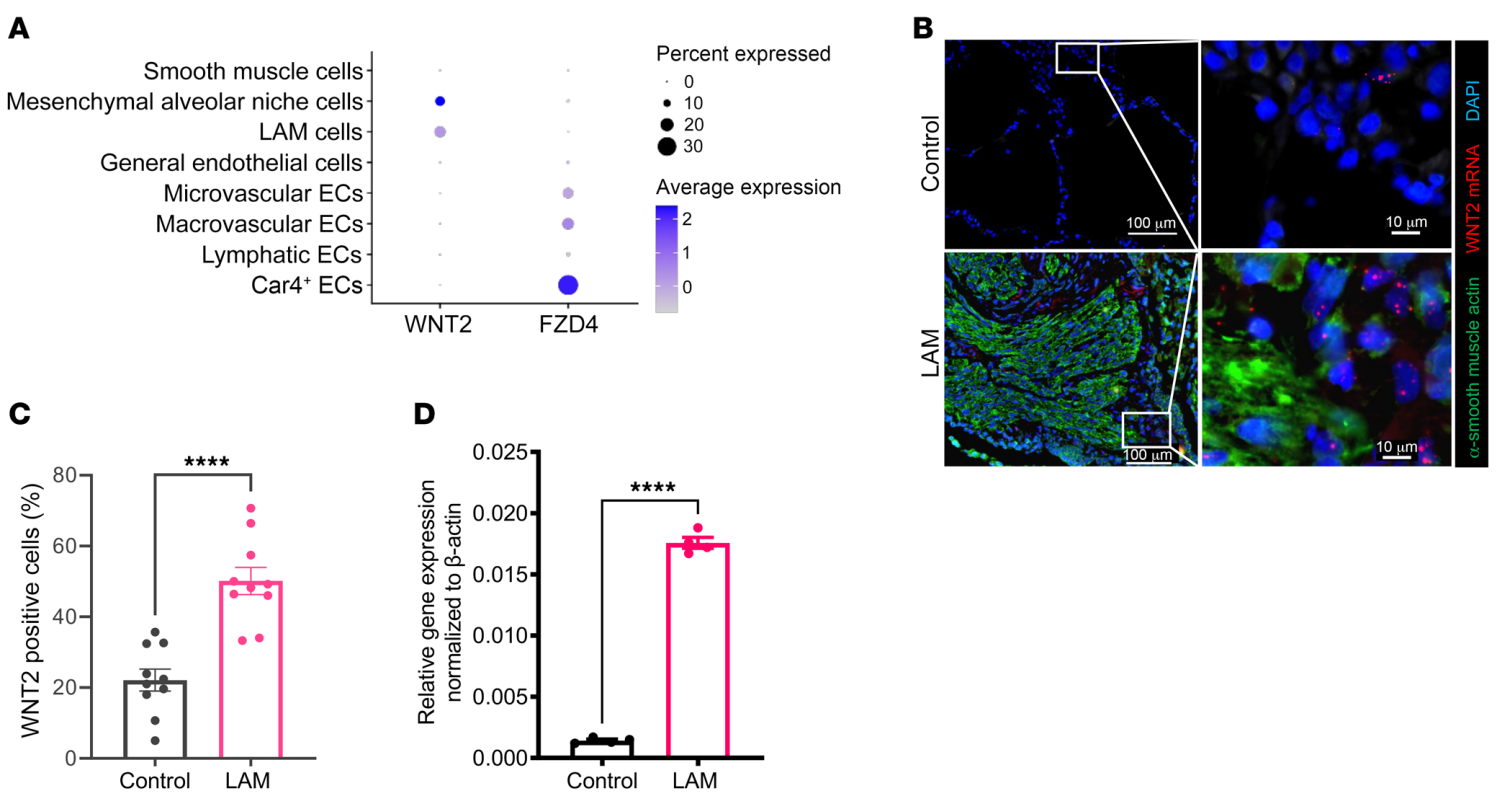
Identification of WNT2 in LAM lung mesenchyme. (**A**) Gene expression dot plot highlighting the unique expression of WNT2 in LAM cell clusters and MANC clusters with corresponding FZD4, a WNT2 receptor, in Car4^+^ EC subclusters within LAM lung. (**B**) Representative images of dual staining with WNT2 mRNAscope (red) ISH and αSMA (green) of LAM (*n* = 3) and control (*n* = 3) human lung. DAPI (blue) shows nuclei. Scale bars: 10 μm and 100 μm. (**C**) Statistical analysis of *WNT2* mRNA–positive cells in control compared with LAM lungs calculated as a percentage of WNT2^+^ cells per total number of cells, as described in Methods. (**D**) Validation of *WNT2* mRNA transcripts in mRNA samples isolated from the lung mesenchyme of control (*n* = 4) and LAM (*n* = 4) lungs. Data points represent relative gene expression values normalized to the expression of the *ACTB* gene using the ΔΔCt method. ****P < 0.0001, by nonparametric *t* test comparisons (**C** and **D**).

In this analysis, we found increased WNT2 ligand expression in 2 mesenchymal cell populations in the LAM lung: mesenchymal alveolar niche cells (MANCs) (32) and unique LAM cells (Figure 3A). We also observed expression of the cognate WNT2 receptor Frizzled 4 (FZD4) in LAM ECs (Figure 3A). Since activation of both canonical and noncanonical WNT signaling pathways play a key role in the preservation of pulmonary vascular homeostasis (33), we confirmed the presence of WNT activation in LAM mesenchymal cells by ISH with RNAscope of the WNT pathway marker AXIN2 (Supplemental Figure 2A). In particular, *WNT2* mRNA was significantly increased in LAM lung fibroblasts compared with expression in healthy human lung fibroblasts by RNAscope of WNT2 (Figure 3, B and C). This was further validated by quantitative PCR (qPCR) for WNT2 in LAM lung fibroblasts compared with control fibroblasts (Figure 3D).

### WNT2 activation of control ECs recapitulates LAM EC morphology.

**Figure 4:**
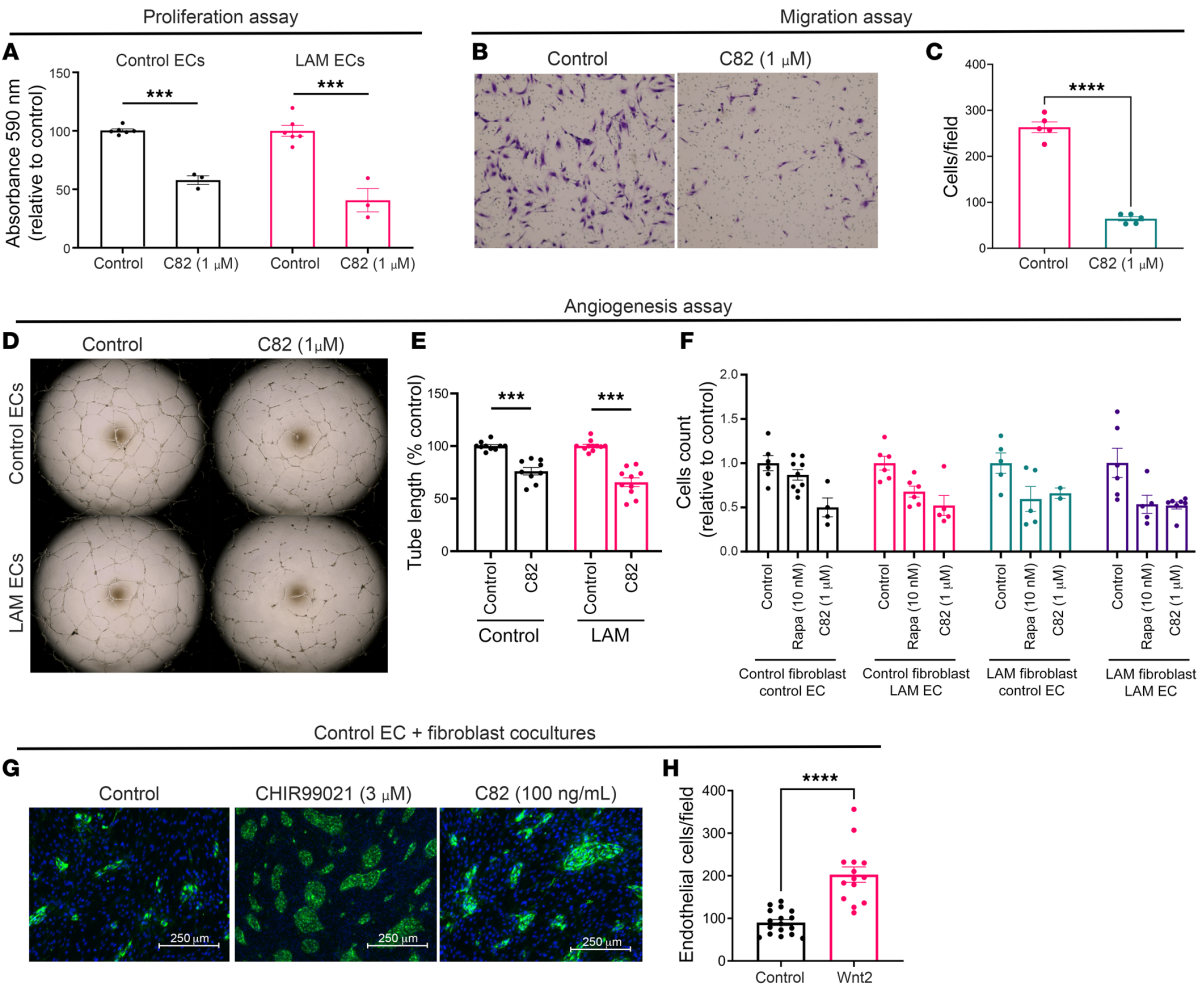
Inhibition of WNT suppresses EC proliferation, migration, and angiogenesis. (**A**) Control ECs (*n* = 6) and LAM ECs cells (*n* = 3) were treated with 1 μM of the WNT inhibitor C82 followed by a proliferation assay as described in Methods. (**B**) Representative images of migration of ECs from LAM lung treated with 1 μM C82 or diluent (control). Original magnification, ×10. (**C**) Statistical analysis of LAM EC migration calculated as the number of cells/field in control (*n* = 5) versus 1 μM C82-treated LAM ECs (*n* = 5). (**D**) Representative images from angiogenesis assay of control ECs and LAM ECs treated with diluent or 1 μM C82. (**E**) Analysis of total tube length of control ECs compared with LAM ECs was performed using the Angiogenesis Analyzer plugin on ImageJ followed by statistical analysis by 2-tailed *t* test, with the control condition taken as 100%. (**F**) Effect of 10 nM rapamycin and 1 μM C82 on EC-fibroblast cocultures. (**G**) EC-fibroblast cocultures of control human ECs with control human lung fibroblasts were treated with diluent (control) and stimulated with the pan-WNT pathway activator CHIR99021 (3 μM) or WNT2 (100 ng/mL). *n* = 3 per treatment group. Scale bars: 250 μm. (**H**) Statistical analysis of ECs per field (with a minimum of 4 images per well) in cocultures treated with WNT2 was performed using a 2-tailed *t* test, and data are presented as the mean ± SEM. ***P < 0.001 and ****P < 0.0001 (**A**, **C**, **E**, and **H**). Nonparametric *t* test (Mann-Whitney) (**A** and **E**).

To assess the effect of WNT pathway inhibition on in vitro EC function, we treated LAM ECs with C82, a WNT/β-catenin pathway transcriptional inhibitor that disrupts the interaction between cyclic AMP response element–binding protein (CBP) and β-catenin (34, 35). C82 inhibition decreased EC proliferation (Figure 4A), migration (Figure 4, B and C), and EC tube formation (Figure 4, D and E). LAM EC cocultures were more susceptible to mTORC1 inhibition than were control EC cocultures, but we observed no significant difference in responses to WNT inhibition (Figure 4F).

When control human ECs cocultured with control human fibroblasts were treated with WNT activators (CHIR99021 or the WNT2 ligand), we noted a change in morphology that recapitulated the LAM EC phenotype and morphology (Figure 4G as compared with Figure 2B). In addition, we observed an increase in EC cell numbers (Figure 4H). Taken together, these experiments demonstrate the ability of control human ECs to adopt LAM EC morphology following WNT pathway activation.

### Loss of Tsc2 in mouse lung mesenchyme causes transcriptomic alterations in ECs.

**Figure 5:**
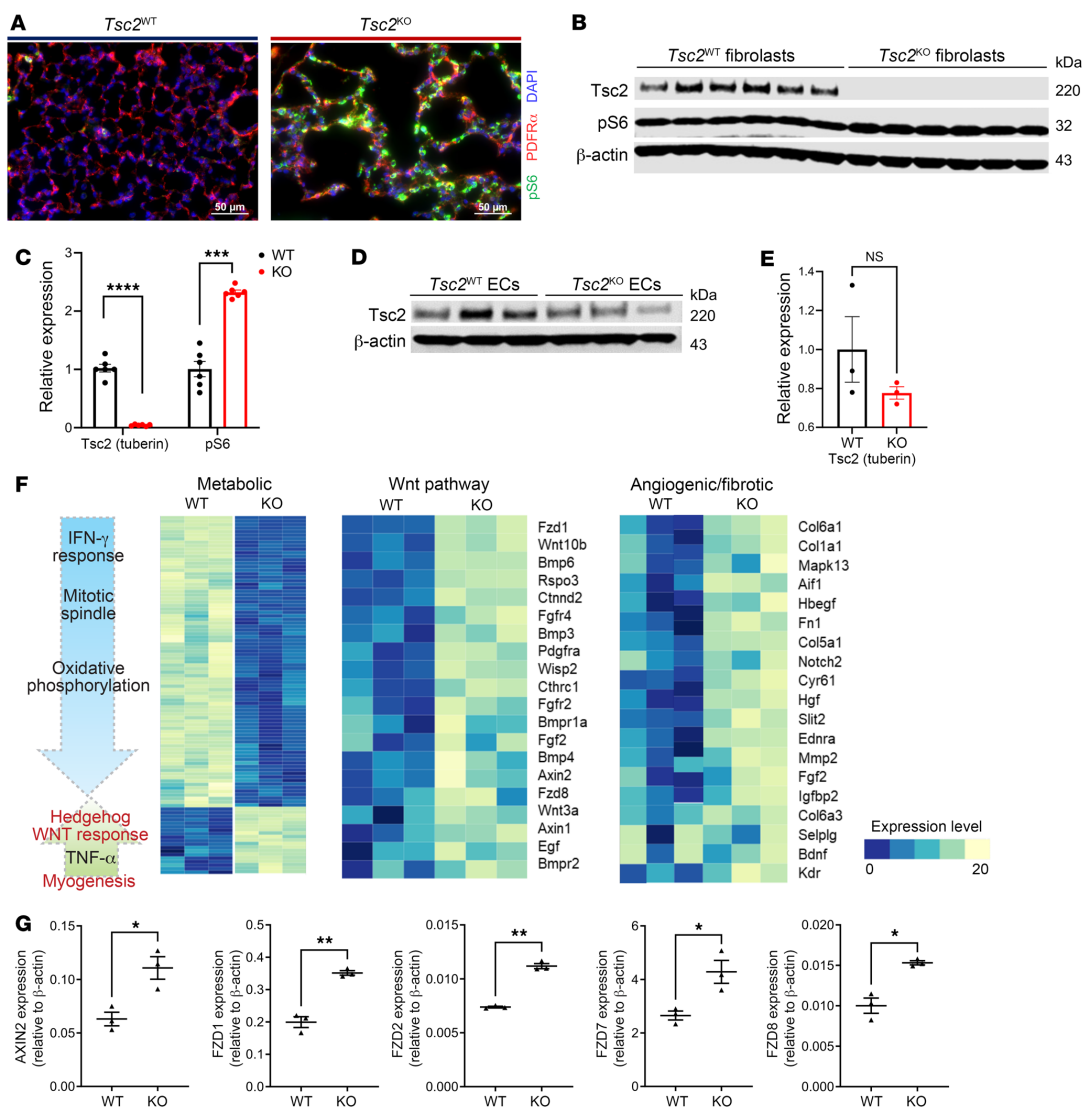
Differential gene expression in CD31^+^ vascular ECs from *Tbx4^LME-Cre^ Tsc2^KO^* mouse lung. (**A**) Representative images of immunofluorescence staining of distal lung demonstrate pS6 positivity, a marker of mTORC1 upregulation (green) in PDGFRα (red) mesenchymal cells of 8-week-old *Tbx4^LME-Cre^ Tsc2^KO^* (Tsc2^KO^) mouse lungs. Scale bars: 50 μm. (**B**) Immunoblot of Tsc2 and pS6 protein expression in *Tsc2*^WT^ (*n* = 3 in duplicates) and *Tsc2*^KO^ (*n* = 3 in duplicates) lung fibroblasts from 12-week-old mice. (**C**) Densitometric analysis of Tsc2 and pS6 levels normalized to β-actin in each lane with average levels in *Tsc2*^WT^ fibroblasts taken as 1. (**D**) Immunoblot of Tsc2 expression in lung ECs from 8-week-old *Tsc2*^WT^ (*n* = 3) and *Tsc2*^KO^ (*n* = 3) mice. (**E**) Densitometric analysis of Tsc2 normalized to β-actin in each lane, with the average expression levels in *Tsc2*^WT^ set as 1. (**F**) Heatmap of the top differentially expressed genes in lung ECs isolated from *Tsc2*^WT^ (*n* = 3) and *Tsc2*^KO^ (*n* = 3) mice. (**G**) qPCR of Wnt pathway activation of *Axin2*, ligands, and receptors in lung ECs from 8-week-old *Tsc2*^WT^ (*n* = 3) and *Tsc2*^KO^ (*n* = 3) mice. Data are presented as the mean ± SEM. *P < 0.05, **P < 0.01, ***P < 0.001, and ****P < 0.0001, by 2-tailed *t* test.

Our data from human LAM samples suggest that mesenchymal LAM cells secrete increased WNT ligands, which may have subsequent effects on adjacent ECs. To test this hypothesis, we used our previously characterized and published *Tbx4^LME-Cre^ Tsc2^fl/fl^* mice (14), a model of mTORC1 hyperactivation with targeted *Tsc2* deletion in lung mesenchymal cells (36). mTORC1 activation was validated by increased pS6 in pulmonary mesenchymal cells from *Tbx4^LME-Cre^ Tsc2^KO^* versus *Tbx4^LME-Cre^ Tsc2^WT^* mice (Figure 5A). Analogous to human LAM disease, cells of mesenchymal lineages in the Tbx4^LME-Cre^ Tsc2^KO^ mice exhibited significant loss of Tsc2 expression with concurrent hyperactivation of mTORC1 detected with pS6 antibody (Figure 5, B and C). Likewise, ECs from *Tbx4^LME-Cre^ Tsc2^KO^* mouse lung expressed Tsc2 at levels comparable to those in ECs from *Tbx4^LME-Cre^ Tsc2^WT^* mice (Figure 5, D and E). We performed bulk RNA-Seq in cells from 8-week-old mice to characterize potential transcriptomic alterations in cellular lineages. Pulmonary ECs from *Tbx4^LME-Cre^ Tsc2^KO^* mice showed marked upregulation in metabolic, growth, and angiogenic pathways compared with lung ECs from *Tbx4^LME-Cre^ Tsc2^WT^* mice (Figure 5F). Analysis of the top differentially expressed genes in *Tbx4^LME-Cre^ Tsc2^KO^* pulmonary ECs showed upregulation of Wnt ligands and amplifiers including *Axin2* and the Wnt receptors *Fzd1*, *Fzd2*, *Fzd7*, and *Fzd8* (Figure 5G). The upregulation of Wnt ligands and amplifiers in ECs from *Tbx4^LME-Cre^ Tsc2^KO^* mice demonstrates activation of the Wnt pathway via crosstalk from mTORC1-activated mesenchymal cells.

### The transcriptome of pulmonary arterial ECs is significantly altered by Wnt signals from mesenchymal cells.

**Figure 6:**
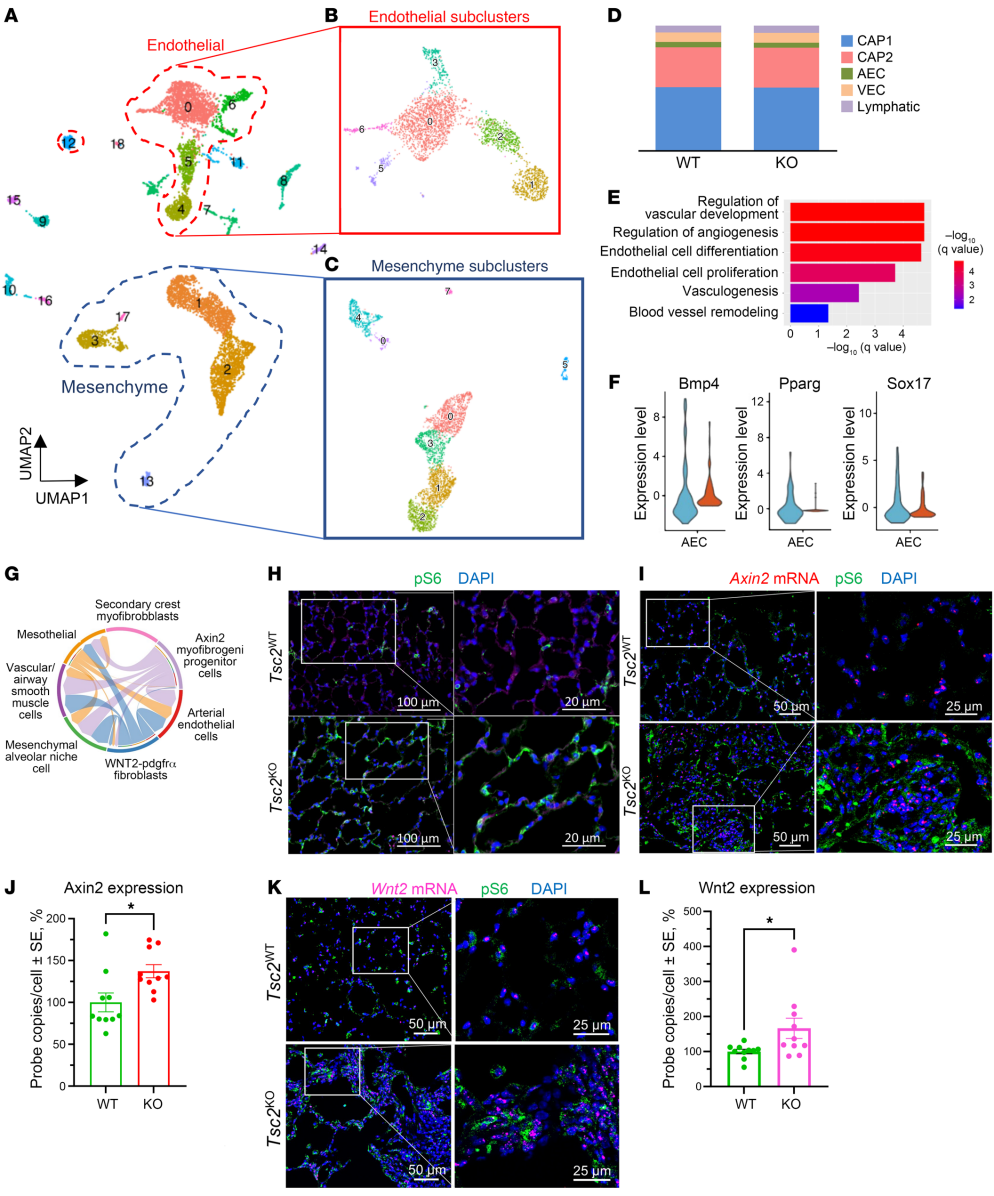
Transcriptomic heterogeneity of ECs in *Tbx4^LME-Cre^ Tsc2^KO^* mouse lung. (**A**) UMAP representation of *Tsc2*^WT^ and *Tsc2*^KO^ mouse lung (*n* = 2) scRNA-Seq, with cell populations labeled according to the corresponding cell type. (**B**) ECs, marked by *Pecam1* and *vWF* expression, were reclustered into 6 subclusters. (**C**) Mesenchymal cells, marked by *Pdgfr*α, *Pdgfr*β and *Msln* expression, were reclustered into 8 subclusters. (**D**) Distribution of each EC type within the EC subclusters. (**E**) GO enrichment analysis of the AEC cluster. (**F**) Violin plot demonstrating increased expression of *Bmp4*, *Pparg*, and *Sox17* in AECs from *Tsc2*^KO^ mice compared with AECs from *Tsc2*^WT^ mice. (**G**) Wnt signaling pathway network (chord diagram) with incoming signal to AECs from 3 mesenchymal cell populations including Axin2 myofibrogenic progenitors, Wnt2-Pdgfrα cells, and mesothelial cells. (**H**) Representative images of 54-week-old *Tsc2*^WT^ (*n* = 3) and *Tsc2*^KO^ (*n* = 3) lung for pS6 (marker of mTORC1 upregulation; magenta); light green reflects an autofluorescence from structural proteins in lung mesenchyme. DAPI (blue) was used to detect nuclei. Scale bars: 20 μm and 100 μm. (**I**) Representative images of dual staining of 54-week-old *Tsc2*^WT^ (*n* = 3) and *Tsc2*^KO^ (*n* = 3) lung for *Axin2* mRNA (magenta) and pS6 (green). Scale bars: 25 μm and 50 μm. (**J**) Quantification of the *Axin2* mRNA data shown in **I**. (**K**) Pulmonary vasculature in both *Tsc2*^WT^ and *Tsc2*^KO^ with *Wnt2* mRNA (magenta) and pS6 (green) from single-molecule fluorescent ISH. Scale bars: 25 μm and 50 μm. (**L**) Quantification of the *Wnt2* mRNA data shown in **K**. Data are presented as the mean ± SEM. *P < 0.05, by 2-tailed *t* test.

We collected and analyzed scRNA-Seq data from 1-year-old *Tbx4^LME-Cre^ Tsc2^WT^* and *Tbx4^LME-Cre^ Tsc2^KO^* mice (Figure 6A). We subclustered EC populations expressing the pan-EC marker *Pecam1* (Figure 6B) and mesenchymal cell populations expressing the markers *Pdgfr*α, *Pdgfr*β, and *Msln* (Figure 6C). Among cells expressing the pan-EC marker *Pecam1*, further clustering generated previously described EC subpopulations including arterial ECs (AECs), VECs, lymphatic ECs, capillary type 1 (CAP1 or general capillary), and capillary type 2 (CAP2 or aerocyte) (Figure 6D). To determine which EC populations were most altered, we performed differential gene expression analysis and gene ontology (GO) analysis comparing each of the EC subpopulations. Compared with other EC populations, AECs were the most transcriptionally altered, with upregulation of pathways associated with vasculogenesis/vasculature development, angiogenesis, EC differentiation, EC proliferation, and blood vessel remodeling (Figure 6E). *Bmp4*, *Pparg*, and *Sox17*, associated with vascular remodeling in PH, were among the genes with the highest differential expression in AECs (Figure 6F). We used CellChat to analyze communication between AECs and each of the mesenchymal cell populations. The top identified interactions included Wnt pathway genes (Supplemental Figure 3) with paracrine signals to AECs from 3 mesenchymal cell populations including (a) Axin2 myofibrogenic progenitors (AMPs), (b) Wnt2-Pdgfrα cells, and (c) mesothelial cells (Figure 6G). ISH of *Tbx4^LME-Cre^ Tsc2^KO^* lungs was notable for concurrent mTORC1 and WNT pathway activation, with increased pS6 (Figure 6H) and *Axin2* mRNA expression (Figure 6, I and J). In addition, we observed increased *Wnt2* mRNA on ISH of *Tbx4^LME-Cre^ Tsc2^KO^* compared with *Tbx4^LME-Cre^ Tsc2^WT^* vessels (Figure 6, K and L).

### mTORC1 activation selectively in lung mesenchymal cells results in pulmonary vascular remodeling, elevated RVSP, and RVH in 1-year-old Tbx4^LME-Cre^ Tsc2^KO^ mice.

Targeted mesenchymal *Tsc2* deletion altered EC angiogenesis, but there were no significant effects on vessel numbers or peripheral muscularization at 12, 16, or 20 weeks of age (Supplemental Figure 4, A and B). However, the time to completion of tube formation was substantially decreased in 8-week-old *Tbx4^LME-Cre^ Tsc2^KO^* ECs (Supplemental Figure 4C). These results indicate that, while there were no histopathological changes at younger ages, ECs isolated from young *Tbx4^LME-Cre^ Tsc2^KO^* mice were functionally altered by the exposure to constitutively activated mTORC1 in the pulmonary mesenchyme.

**Figure 7:**
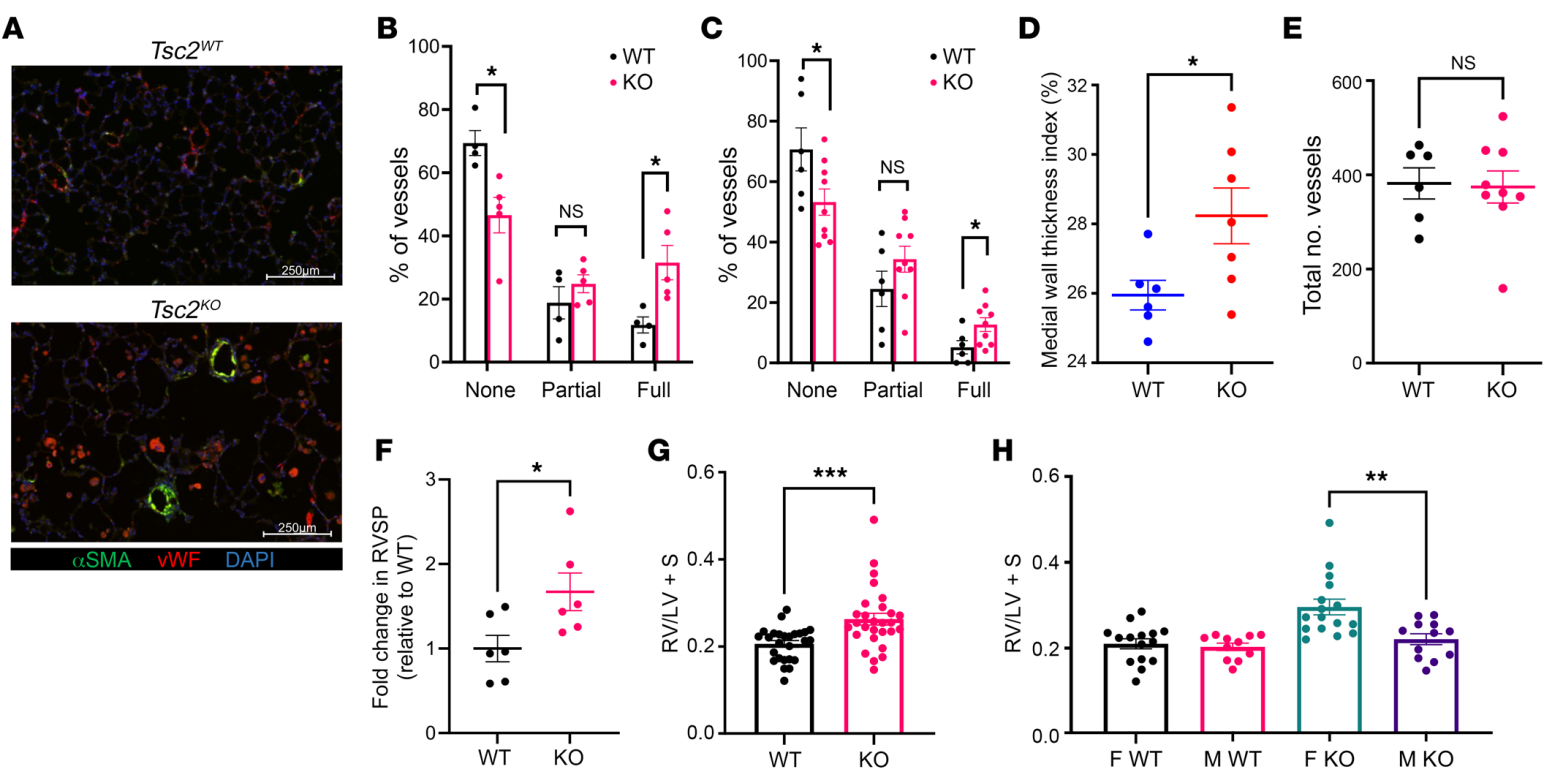
Pulmonary vascular remodeling and right heart dysfunction in 1-year-old *Tbx4^LME-Cre^ Tsc2^KO^* mice with mesenchymal mTOR activation. (**A**) Representative images of vessels in *Tsc2^WT^* compared with *Tsc2^KO^* in 1-year-old mice. Scale bar: 250 μm. (**B**) Scoring of peripheral muscularization based on 15 randomly acquired images per mouse (*n* = 9). (**C**) Peripheral muscularization based on automated measurements obtained on Visiomorph. Morphometric analysis of pulmonary vascular remodeling in *Tsc2^KO^* (*n* = 9, red) mice compared with *Tsc2^WT^* mice (*n* = 6, blue). (**D**) Medial wall thickness based on Visiomorph. Medial wall thickness was defined as: (vessel diameter minus luminal diameter)/2. Comparison of *Tsc2^WT^* (*n* = 6) to *Tsc2^KO^* (*n* = 7). (**E**) Total number of vessels unchanged in *Tsc2^WT^* versus *Tsc2^KO^*. (**F**) Right heart catheterization of 1-year-old *Tsc2^WT^* (*n* = 6) and *Tsc2^KO^* (*n* = 6) mice with comparison of RVSP in terms of fold change relative to baseline *Tsc2^WT^* RVSP of 12.9 mmHg. (**G**) RVH as measured by the Fulton index (RV/S + LV); *Tsc2^WT^* (*n* = 25) versus *Tsc2^KO^* (*n* = 27). (**H**) Increased Fulton indices were driven by female *Tsc2^KO^* mice (*n* = 13) compared with male *Tsc2^KO^* mice (*n* = 11). Data are presented as the mean ± SEM. *P < 0.05, **P < 0.01, and ***P < 0.001, by 2-tailed *t* test.

To determine whether prolonged exposure to *Tsc2^KO^* mesenchyme would further alter EC function and vascular morphology, we reassessed the pulmonary vasculature in 54-week-old mice. Analyses of 54-week-old *Tbx4^LME-Cre^ Tsc2^KO^* lungs (Figure 7A) revealed significant vascular remodeling compared with age-matched *Tbx4^LME-Cre^ Tsc2^WT^* control lungs (Figure 7, B and C). This was characterized by an increase in fully muscularized vessels with a subsequent decrease of nonmuscularized vessels, which was confirmed using manual counts of selective immunohistochemical staining of vessels (Figure 7B) and an automated analysis using a program that identifies vessels and calculates degree of peripheral vascularization (Visiomorph) (Figure 7C). In addition, pulmonary vessels from *Tbx4^LME-Cre^ Tsc2^KO^* mice had thicker medial walls (Figure 7D and Supplemental Figure 5), however, there were no differences in the total number of vessels (Figure 7E). We investigated RVSPs in 54-week-old mice by right heart catheterization and found elevated ventricular pressures in *Tbx4^LME-Cre^ Tsc^KO^* mice compared with *Tbx4^LME-Cre^ Tsc2^WT^* mice (Figure 7F). *Tbx4^LME-Cre^ Tsc2^KO^* mice also had larger hearts, as measured by gross weight, with higher RV mass, as measured by the Fulton index (Figure 7G). The Fulton index, a metric of RV hypertrophy (RVH), showed that RVH was most notable in female *Tbx4^LME-Cre^ Tsc2^KO^* mice (Figure 7H). These findings were not present in mice at younger ages (Supplemental Figure 6). Taken together, these findings demonstrated that mesenchymal mTORC1 activation resulted in spontaneous vascular remodeling in 1-year-old *Tbx4^LME-Cre^ Tsc2^KO^* mice.

## Discussion

The study of pulmonary vascular remodeling has been limited by disease heterogeneity due to polygenic contributions. Monogenic diseases such as LAM offer a unique opportunity to study the role of specific signaling pathways in the initiation and progression of vascular remodeling. However, as vascular ECs from LAM lungs do not harbor mutations, it was previously unclear how mesenchymal LAM cells with constitutive mTORC1 hyperactivation altered EC function and could contribute to vascular remodeling. Using human LAM samples and a murine model, our study identifies cell crosstalk in the LAM lung by demonstrating that hyperactive mTORC1 mesenchyme orchestrated transcriptomic and functional changes in ECs. This contributed to the development of pulmonary vascular remodeling through paracrine stimulation of ECs by *TSC2*-null LAM mesenchymal cells. In the presence of hyperactivated mesenchymal mTORC1, vascular ECs from LAM lungs exhibited EC dysfunction characterized by altered proliferation, migration, and angiogenesis. These data suggest that EC reprogramming in LAM occurred as a result of paracrine ligand production by mesenchymal cells and receptor activation on ECs, which we believe defines a novel mesenchymal cell/EC axis in the LAM lung. Moreover, parallel to the development of PH in a subset of patients with late-stage LAM, mesenchymal mTORC1 hyperactivation in aged *Tbx4^LME-Cre^ Tsc2^fl/fl^* mice led to spontaneous development of pulmonary vascular remodeling, highlighting the pathogenic importance of mTORC1 activation in the mesenchymal cell/EC axis.

Our study adds to emerging evidence that mTOR signaling contributes to the development of pulmonary vascular remodeling through pulmonary vascular cell proliferation, vasoconstriction, and chronic hypoxia (15). To date, much of the existing evidence for the effect of aberrant mTOR signaling on pulmonary vascular remodeling is limited to experimental murine models and in vitro studies utilizing primary pulmonary vascular cells. Our research focused on ECs because of their crucial role in PH initiation and progression, whereas previous studies of mTORC1 activation and pulmonary vascular remodeling have largely focused on PASMCs (37, 38), where reduced TSC2 in PASMCs is associated with hyperproliferation, remodeling, and PH (39). Recent research on pulmonary vascular remodeling suggests a phenotype shift in PASMCs from contractile to synthetic phenotypes as well as skewed cellular interaction in PH samples, with most cellular interactions shifted toward smooth muscle cells and fibroblasts (40). However, ECs have been shown to play a crucial role in pulmonary vascular remodeling through alterations in vascular tone, metabolism, oxidative stress, cell growth, and differentiation (41). Moreover, ECs can trigger PASMC proliferation through paracrine signaling (42). In our study, we found that ECs were both transcriptomically and functionally altered in the presence of hyperactive mTORC1 mesenchymal cell populations.

This phenomenon may not be unique to LAM, as it has been shown that primary pulmonary ECs isolated from patients with PH are hyperproliferative, with increased migration but reduced angiogenic capacity on standard angiogenesis assays (25, 26). However, how these ECs are affected by other pulmonary cell types and paracrine ligand/receptor signaling remains unclear. Specifically, it is unclear whether this pattern of hyperproliferative potential but reduced angiogenic capacity is a true feature of ECs or whether this is secondary to limitations of in vitro assays where ECs are neither exposed to the paracrine signaling nor experiencing biomechanical forces from the pulmonary ECM. To determine the effects of support cells and biochemical forces on the angiogenic capacity of LAM ECs, we developed EC-fibroblast cocultures to interrogate cell-cell interactions and 3D EC-fibroblast cocultures to investigate cell-matrix interactions. We found that LAM ECs, when cocultured with fibroblasts, had markedly enhanced growth and substantially altered cellular segregation, with further augmentation of EC tube length in 3D matrix models.

To further understand the mechanisms of endothelial dysfunction in an mTORC1 hyperactivated mesenchymal state, we used a murine model of selective mTORC1 activation in pulmonary progenitor mesenchymal cells. In *Tbx4^LME-Cre^ Tsc2^KO^* mice, we showed that transcriptomic upregulation of metabolic, growth factor, and angiogenic genes in ECs occurred by 8 weeks of age, before phenotypic or histopathological changes were observed. However, ECs isolated from *Tbx4^LME-Cre^ Tsc2^KO^* mice at this age were functionally altered by exposure to constitutively activated mTORC1 in pulmonary mesenchyme. In fact, *Tbx4^LME-Cre^ Tsc2^KO^* mice did not exhibit spontaneous pulmonary vascular remodeling with elevated right heart pressures or RVH until 1 year of age. Intriguingly, this is comparable to a human age of approximately 40–50 years (43, 44), the average age of respiratory failure in patients with LAM requiring initiation of the allosteric mTOR inhibitor rapamycin (sirolimus) (45). In addition, the mild-to-modest hemodynamic alteration observed in the murine model mirrors the mild PH in patients with LAM (7). As such, the development of spontaneous vascular remodeling in our aged transgenic mice is analogous to the development of PH in end-stage LAM disease and provides a mechanistic link between constitutive mesenchymal mTORC1 activation and the development of pulmonary vascular remodeling.

The presence of transcriptomic changes prior to the onset of pulmonary vascular remodeling in our murine model offered a unique opportunity to study ECs in a pre-disease state and highlights the importance of EC subpopulations. Next-generation sequencing allowed us to characterize mechanistic pathways in an agnostic fashion, leading to identification of the WNT/β-catenin pathway as an activator of EC dysfunction in the presence of mesenchymal mTORC1 activation. Transcriptomic profiling of pulmonary cell populations in PH is notable for EC heterogeneity (46). Different EC clusters revealed changes in cell regulation essential for the pathogenesis of PH, including upregulation of EIF2 and mTORC1 signaling (47). We identified the Wnt ligand WNT2 as a Wnt pathway signaling protein expressed by LAM mesenchyme. The canonical Wnt pathway is known to determine the vasculogenic fate of postnatal mesenchymal stem cells (48) with EC differentiation (49) and angiogenesis (50) contingent on WNT/β-catenin signaling. Within the lung, Wnt signaling has been demonstrated to regulate the microvascular niche and drive adaptive angiogenesis in response to injury (51). We showed here that WNT inhibition limited proliferation, migration, and angiogenesis of ECs from LAM lung. The signaling between ECs and fibroblasts in cocultures was inhibited by blocking either WNT/β-catenin or mTORC1 signaling. Last, WNT2 activation of ECs from control human lung elicited a LAM-like phenotype in vitro, characterized by increased cell numbers and altered cellular morphology. These findings validate the importance of the WNT pathway in pulmonary EC function and highlight the therapeutic potential of WNT pathway targets.

Taken together, our research demonstrates that mesenchymal cells with hyperactive mTORC1 activity drive EC dysfunction in LAM and in a murine model of mesenchymal mTORC1 activation. Recent research indicates complexity and heterogeneity of ECs in the pulmonary vasculature (42). Further studies are needed to delineate the significance of specific EC and mesenchymal cell subpopulations involved in EC dysfunction, their responses to physiological and pathological stimuli, and their contributions to pulmonary vascular homeostasis in health and disease. Our study in LAM and the murine model of targeted *Tsc2* deletion in lung mesenchymal cells highlights paracrine activation of receptors on ECs and reprogramming of EC functions, demonstrating the prominent pathogenic importance of this cell-cell communication axis. This may have wider implications for human health, as a growing body of literature has suggested that mTOR has an important role in the pathogenesis of pulmonary diseases including pulmonary arterial hypertension (15, 17–19), chronic obstructive pulmonary disease (52), and idiopathic pulmonary fibrosis (53, 54).

## Methods

### Experimental animals.

As previously described (14), *Tsc2^loxP/loxP^* mice (55) were crossed with *Tbx4^LME-Cre^* mice (36).

### Mouse lung histology and morphometry preparation.

Lungs were inflated under constant pressure (25 cm H_2_O), and the pulmonary circulation was flushed with 2 cc PBS as described previously (14, 56).

### Human lung tissue.

Normal lung samples were obtained through the University of Pennsylvania Lung Biology Institute’s Human Lung Tissue Bank. These samples were obtained from deceased donors and represent a secondary use of tissue. LAM lung samples were obtained from living donors at the time of lung transplantation through the National Disease Research Interchange (NDRI) (Philadelphia, Pennsylvania, USA). Informed consent was obtained by the NDRI prior to acceptance of tissue donation for research. For both healthy and diseased (LAM) lung samples, identifying information was removed prior to use in accordance with institutional and NIH protocols.

### Tissue and scRNA-Seq sample preparation.

Lung parenchymal tissue preparation and scRNA-Seq sample preparation were performed as previously described (23, 24). In brief, human lungs specimens were first confirmed to be from the most distal portion of the lung through direct visualization of the pleural lining. The pleural lining was separated from the lung parenchyma and discarded followed by microdissection along the bronchovascular tree. Specimens were then mechanically minced prior to enzymatic digestion with collagenase, dispase and DNase. Samples were then filtered, RBC lysed with ACK lysis buffer,and processed into a single-cell suspension. CD45^+^ immune cells were depleted using MACS LS columns with CD45 microbeads (Miltenyi Biotec, 130-045-801) with 2 × 10^6^ cells per column to enhance purity and viability. Following the single-cell suspension, the samples were loaded onto a GemCode instrument (10X Genomics) to generate single-cell barcoded droplets (GEMs) according to the manufacturer’s protocol.

### Immunohistochemistry.

Human and murine paraffin-embedded tissue sections used for the immunohistochemistry experiments were sectioned and deparaffinized in xylene followed by isopropanol dilutions. Antigen retrieval was performed with 10 mM sodium citrate buffer (10 mM sodium citrate, 0.05% Tween 20, pH 6.0) following staining with primary antibodies at 4°C overnight. Secondary antibodies were incubated for 60 minutes at room temperature (22°C–24°C). Slides were mounted with Faramount Aqueous Mounting Media (Dako, S3025). See Table 1 for the list of primary and secondary antibodies used.

### RNAscope assay.

Human paraffin-embedded tissue sections were deparaffinized according to standard procedures prior to start of RNAscope protocol. Target mRNA was detected using the RNAscope Multiplex Fluorescent Reagent Kit, version 2 (Advanced Cell Diagnostics, 323100) according to the manufacturer’s manual. For visualization of the probe’s hybridization, we used Opal 570 (Akoya Biosciences, SKU: FP1488001KT) and Opal 690 (Akoya Biosciences, SKU: FP1497001KT) fluorophores. Images were captured on a Nikon Eclipse 2000 microscope and a Leica Stellaris 5 laser confocal microscope.

### RNAscope analysis.

To obtain the average ratio of RNAscope probe copy to cell, 10 images from each sample were analyzed. Images were analyzed using the “analyze particles” plugin in Fiji 1.53.t software. Minimal and maximal particle sizes for the plugin were obtained from direct measurements of both the RNAscope fluorescent probe spot minimal size and the range of the nucleus size. The numbers of RNAscope probe copy in aggregates were counted by dividing the aggregate area by the minimal spot size. Data were normalized to the control.

### Morphological analysis.

To quantify the frequency and severity of pulmonary vascular changes in transgenic mice, whole sections of mouse lungs were stained with α-smooth muscle actin (αSMA) (MilliporeSigma, 2547) and vWF (Dako, A0082) using standard immunohistochemical protocols. Scoring of peripheral muscularization was based on 15 randomly acquired images per mouse. Peripheral vessels were defined as those small vessels distal to the terminal, muscularized bronchioles. Using a previously described method (57), the degree of muscularization was defined by αSMA positivity around the vascular walls and classified as nonmuscularized (<20%), partially muscularized (20%–70%), and fully muscularized (>70%). In addition, sequential mouse lung specimens were stained with αSMA and vWF, with automated whole lung analysis of vasculatures performed by Visiomorph software (Visiopharm).

### Isolation of primary ECs and fibroblasts.

Single-cell suspensions from distal human and whole mouse lungs were prepared using previously described methods (23, 24). Immune cells were depleted using CD45 microbeads (Miltenyi Biotec, 130-045-801), and ECs were isolated after CD31^+^ selection with CD31^+^ microbeads (Miltenyi Biotec, 130-091-935). ECs were grown on EGM2 (Lonza, CC3162) on collagen-coated flasks. Fibroblasts were derived from the CD45^–^CD31^–^ fraction that was subsequently depleted of epithelial cells using EPCAM-conjugated Dynabeads (Thermo Fisher Scientific, 1120D). ECs were grown to confluence and sorted a second time with CD31^+^ microbeads prior to experimentation in passages 2 to 4.

### Proliferation assay.

Primary cells between passages 2 and 4 were seeded in 96-well plates at 2.5 × 10^3^ cells/well in 200 μL media with 2.5% FBS. Cells were fixed with 25% glutaraldehyde and stained with 0.05% crystal violet (MilliporeSigma, C3886). Cells were lysed with methanol prior to absorbance reading at 590 nm. For treatments, compounds were added on the day after plating and further incubated for 48 hours or 72 hours. The WNT inhibitor C82 was dosed at 1 μM (dose was selected on the basis of the IC_50_ of control cells).

### Transwell cell migration assay.

Transwell inserts (8 μm pores, Corning, 353097) were transferred onto 24-well plates with culture medium. Cell suspensions were added to the upper chamber and then incubated at 37°C and 5% CO_2_ for 20 hours. Cells were fixed with glutaraldehyde and stained with crystal violet. Images were captured with an EVOS cell imaging camera (Life Technologies, Thermo Fisher Scientific), and the number of cells/field was manually counted.

### EC-fibroblast coculture (2D monolayer).

Fibroblasts were plated on 12-well plates at 9 × 10^4^/well and incubated for 36 hours prior to the addition of ECs at 3 × 10^4^/well. Media (10% EBM plus EGM2 without heparin) were changed every 2 days, and the cocultures were grown for 6 additional days prior to fixation (2% paraformaldehyde [PFA]) and staining.

### ECs in Matrigel (3D spherical).

Wells within a 12-well plate were precoated with fibronectin (Thermo Fisher Scientific, 33016015). Primary ECs, isolated as above, were collected between P2 and P4 and resuspended at 5 × 10^3^ cells in 50 μL EGM2 (Lonza). Matrigel (50 μL) was added to each sample. Each 100 μL EC-Matrigel mixture was dispensed as a droplet onto the middle of the well followed by incubation at 37°C for 30 minutes to induce polymerization. Prewarmed 2 mL EGM2 was added to each well with media changes every 2 days.

### EC-fibroblast cocultures in Matrigel (3D spherical).

Wells within a 12-well plate were precoated with fibronectin (Thermo Fisher Scientific, 33016015). Primary ECs and fibroblasts isolated as above were collected between P2 and P4. A mixture of 5 × 10^3^ ECs and 1.5 × 10^4^ fibroblasts were resuspended in 50 μL of 50% EGM2/50% DMEM. Matrigel (50 μL) was added to each sample. Each 100 μL EC-fibroblast coculture plus Matrigel mixture was dispensed as a droplet onto the middle of the well followed by incubation at 37°C for 30 minutes to induce polymerization. Prewarmed 50% EGM2/50% DMEM (2 mL) was added to each well, with media changes every 2 days.

### RNA isolation and qPCR analysis for gene expression.

The RNeasy Micro Kit (QIAGEN) was used to isolate RNA according to the manufacturer’s protocol. Determination of RNA concentration and purity was performed by OD measurement (ratio of OD at 260 nm to OD at 280 was >1.7) using a NanoDrop spectrophotometer (Thermo Fisher Scientific). cDNA was synthesized using the Verso cDNA synthesis kit (Thermo Fisher Scientific). qPCR was performed using Fast SYBR green reagents on the QuantStudio 7 Thermocycler (Life Technologies, Thermo Fisher Scientific).

### Immunoblot analysis.

Cells were washed with DPBS prior to lysis on ice for 15 minutes in RIPA cell lysis buffer (MilliporeSigma) supplemented with protease and phosphatase inhibitors (Roche) as described before (14, 58). Samples were separated by SDS-PAGE, transferred onto nitrocellulose, blocked, and incubated overnight at 4°C with the diluted primary antibody. Blots were washed and then incubated for 1 hour at room temperature with the appropriate LI-COR Biosciences secondary antibody. Image acquisition and band intensity quantification were performed using the Odyssey IR imaging system (LI-COR Biosciences). Primary antibodies against TSC2, β-actin, and pS6 were purchased from Cell Signaling Technology.

### Analysis of scRNA-Seq data.

Reads were aligned using STARsolo version 2.7.9a. For further processing, integration, and downstream analysis, Seurat version 4 was used. Cells that express fewer than 500 genes and/or genes with a greater than 2 median absolute deviation above the median were filtered out. Cells that had mitochondria counts higher than 10% were also removed. The cell-cycle phase score was calculated using the CellCycleScoring function in Seurat, and data were normalized and scaled using the SCTransform function, regressing out the effects of the cell cycle, percentage of mitochondria, number of unique molecular identifiers (UMIs) per cell, and number of features per cell.

Integration of the individual samples was performed using the normalized counts from SCTransform and the top 3,000 variable genes as anchors. Linear dimension reduction was done using principal component analysis (PCA), and the number of PCA dimensions was evaluated and selected on the basis of the ElbowPlot assessment. Data were clustered using the Louvain graph–based algorithm in Seurat at an appropriate cluster resolution. The uniform manifold approximation and projection (UMAP) dimension reduction algorithm was used to project the cells onto a 2D space for visualization. The cellular compartments (endothelium, epithelium, mesenchyme, and immune) were identified using known canonical marker genes. Following compartment identification, the endothelial and mesenchymal clusters were subsetted out, and the clustering and dimension reduction steps were repeated. The cell populations were then identified and annotated using either known canonical marker genes or by the assessment of the cluster-defining genes based on differential expression using the FindMarkers function in Seurat version 4. GO analysis was performed using the ClusterProfiler package in R. Intercellular network analysis was performed using CellChat version 1.6.1. The minimum cell count for filtering was set at 10, and only cell populations of interest were selected for the analysis.

### Hemodynamics measurements (right heart catheterization).

Hemodynamics studies were performed by the University of Pennsylvania Rodent Cardiovascular Phenotyping Core (RRID: SCR_022419) at the University of Pennsylvania. Mice were anesthetized by inhalation of 2% isoflurane. The right lateral neck was dissected to isolate the right internal jugular vein. A high-fidelity pressure catheter (Millar, SPR671NR) was introduced into the right internal jugular vein and passed into the right ventricle for intraventricular arterial pressure monitoring. RVSP, end-diastolic pressure, heart rate, and rate of pressure change (dP) with time (dt) during isovolemic contraction were recorded and analyzed using a PowerLab 10 (AD Instruments). After data acquisition, the pressure catheter was removed, and the animals were euthanized.

### Measurement of RVH (Fulton index).

Hearts were excised and the RV free wall was dissected along the interventricular septum. RVH was calculated as the weight ratio of the RV free wall relative to the left ventricle plus the septum (RV/LV + S). Measurements were captured using wet weights as well as dry weights, with the latter obtained after specimens were dehydrated at 55°C overnight.

### Statistics.

Statistical analyses were performed in GraphPad Prism 9 (GraphPad Software). A 2-tailed Student’s *t* test was used for comparisons between 2 experimental groups with a Shapiro-Wilk test for normality. For experiments with more than 2 groups, a nonparametric Kruskal-Wallis ANOVA was performed with Siegel (Bonferroni’s) correction for post hoc pairwise contrasts. Statistical data were considered significant when *P* was less than 0.05.

### Study approval.

All mouse experiments were performed under protocols approved by the guidance of the IACUC of the University of Pennsylvania and conformed to NIH guidelines on animal care. Human LAM specimens were obtained from the NDRI at the time of lung transplantation through established protocols. The normal human samples used in this study were from deidentified, nonused lungs donated for research (PROPEL, approved by University of Pennsylvania IRB), with informed consent, in accordance with institutional and NIH procedures. Consent was provided by next of kin or health care proxy, and all patient information was removed before use. This use does not meet the current NIH definition of human subject research, but all institutional procedures required for human subject research were followed throughout the reported experiments.

### Data availability.

Murine lung scRNA-Seq data that support the findings of this study have been deposited in the NCBI’s public functional genomics data repository Gene Expression Omnibus (accession GSE249634). Values for all data points in graphs can be found in the Supplemental Supporting Data Values file. Any remaining data supporting the results of the study will be made available from the corresponding author upon reasonable request. All unique human and mouse lung cell lines generated in this study are available from the corresponding author under a material transfer agreement.

## Author contributions

SML designed and performed experiments, developed 2D and 3D EC cocultures systems, performed IHC and immunofluorescence analyses, collected and measured Fulton index data, interpreted the data, and wrote the manuscript. RR assisted with mouse care as well as mouse lung isolation. SML, MCB, LTF, JDP, ANN, EC, and EEM provided access to human samples and assisted with tissue processing. AG, CJS, and OAL assisted with cell culture experiments. SC assisted with hemodynamic measurements, and with GK, performed Visiomorph analysis. KO processed mouse lung tissue for bulk RNA-Seq and assessed vessel wall thickness. AB provided bioinformatic support for analysis of the single-cell data set. ARM performed multiple-channel IHC and RNAscope staining and image acquisition and analysis. KSV designed qPCR primers and performed the qPCR experiments. JFE performed Western blot analysis and helped write the manuscript. VPK designed the study, interpreted the data, wrote the manuscript, and directed the project.

## Supplementary Material

Supplemental data

Unedited blot and gel images

Supporting data values

## Figures and Tables

**Table 1:**
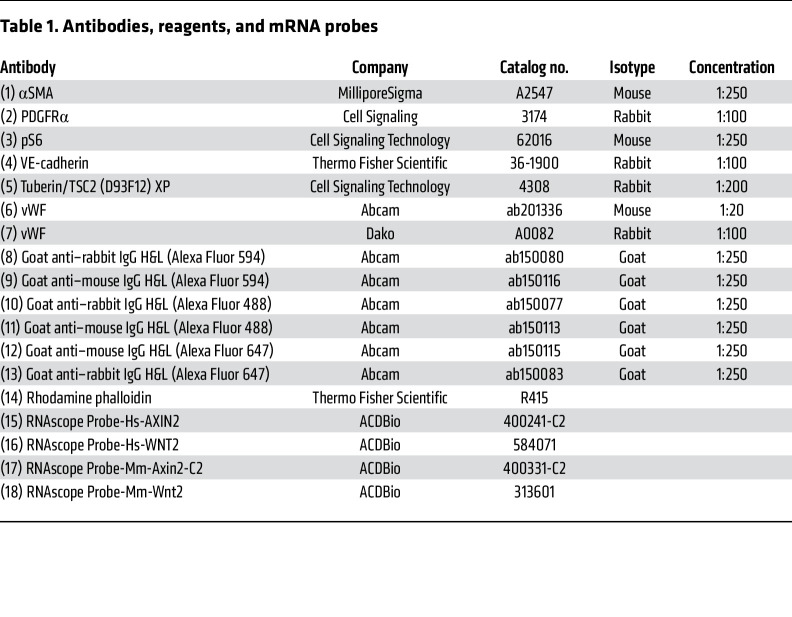
Antibodies, reagents, and mRNA probes

## Version 1 12/21/2023

In-Press Preview

## Version 2 02/15/2024

Electronic publication
