# Supplementary material for: Hyperactive mTORC1 in lung mesenchyme induces endothelial cell dysfunction and pulmonary vascular remodeling
Source: J Clin Invest. 2023 Dec 20;134(4):e172116. doi: 10.1172/JCI172116 (PMC10866655; doi:10.1172/JCI172116)
Supplement: Supplemental data [file jci-134-172116-s041.pdf]

Supplemental Figures

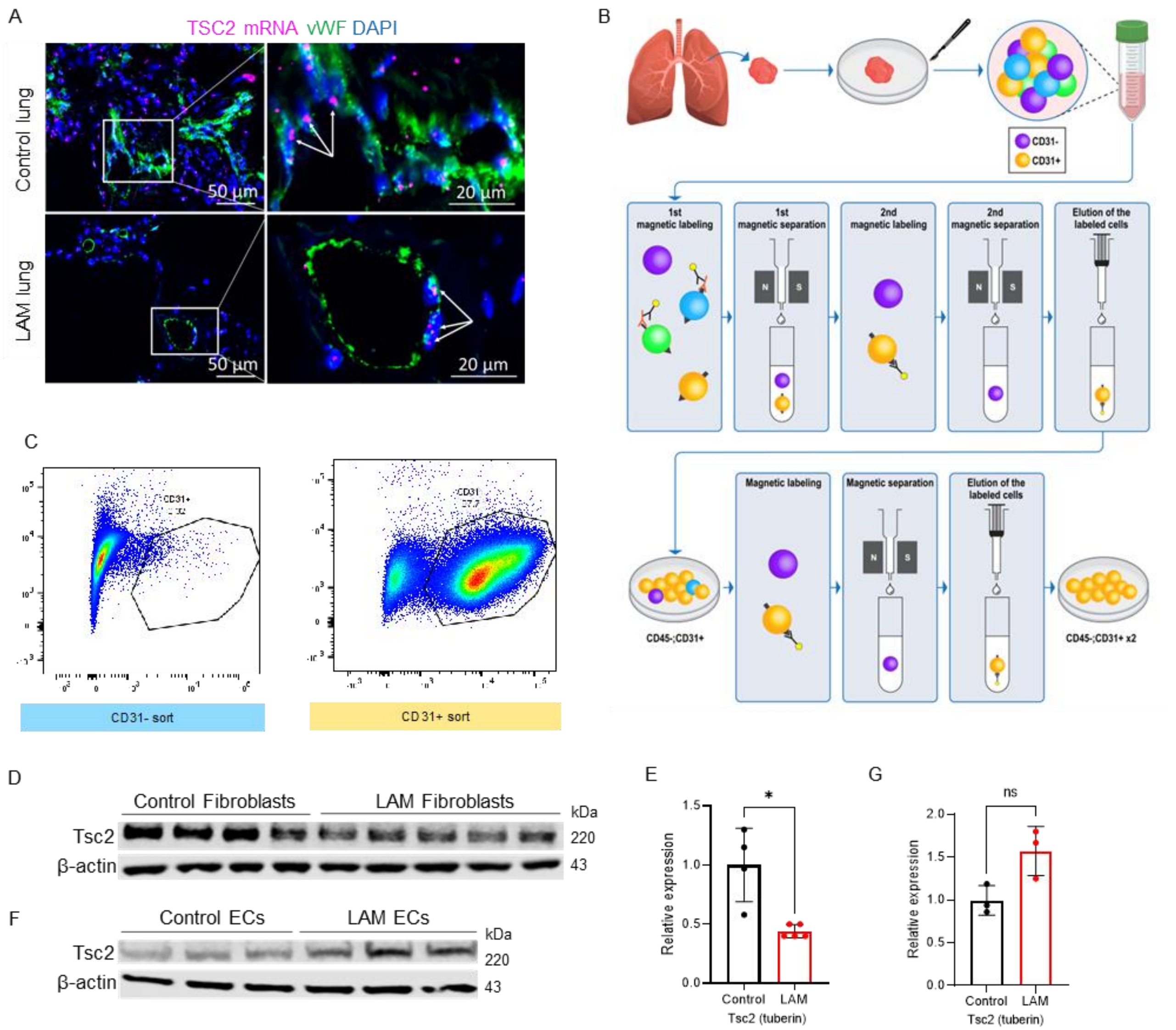

Supplemental Figure 1. Isolation, purification, and characterization of pulmonary ECs from lung explants of patients with LAM. (A). Representative images of dual staining of human control lung and LAM lung to detect TSC2 RNA using RNAscope (turquoise) and immunostaining for vWF (green); DAPI (blue) detects nuclei. (B) Schematic representation of the procedure used for ECs isolation. (C) Confirmation of purity of primary ECs isolates by flow cytometry of CD31+ sorted cells grown in primary cell culture with high enrichment (87.7%) versus CD31-depleted cells (0.32% positive for CD31). (D) Immunoblot analysis of TSC2 expression in lung fibroblasts from control (n=4) and LAM (n=5) lungs. (E) Statistical analysis of TSC2 densitometry normalized to β-actin with the average expression levels in control fibroblasts as 1. (F) Immunoblot analysis of Tsc2 expression in lung ECs from control human (n=3) and LAM (n=3) lungs. (G) Statistical analysis of TSC2 densitometry normalized to β-actin with the average expression levels in control ECs as 1. Significance and SD (C and D) was determined by students T test. ns=non-significant; * P< 0.05.

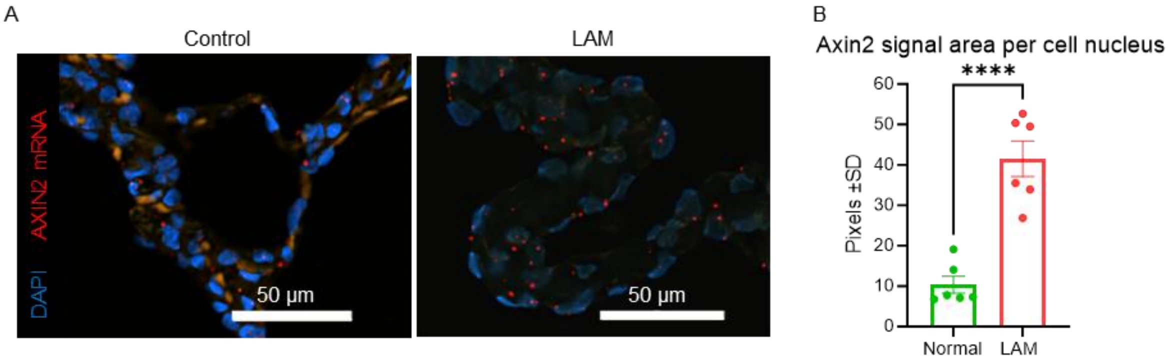

Supplemental Figure 2. WNT activation in LAM lungs. (A) AXIN2 In Situ Hybridization using RNA-scope in LAM and control human lung. AXIN2 mRNA was detected using RNAScope probe (NM_004655.3, target region 502-1674, Advanced Cell Diagnostics) and visualized with Opal 570 fluorophore (Akoya Biosciences).

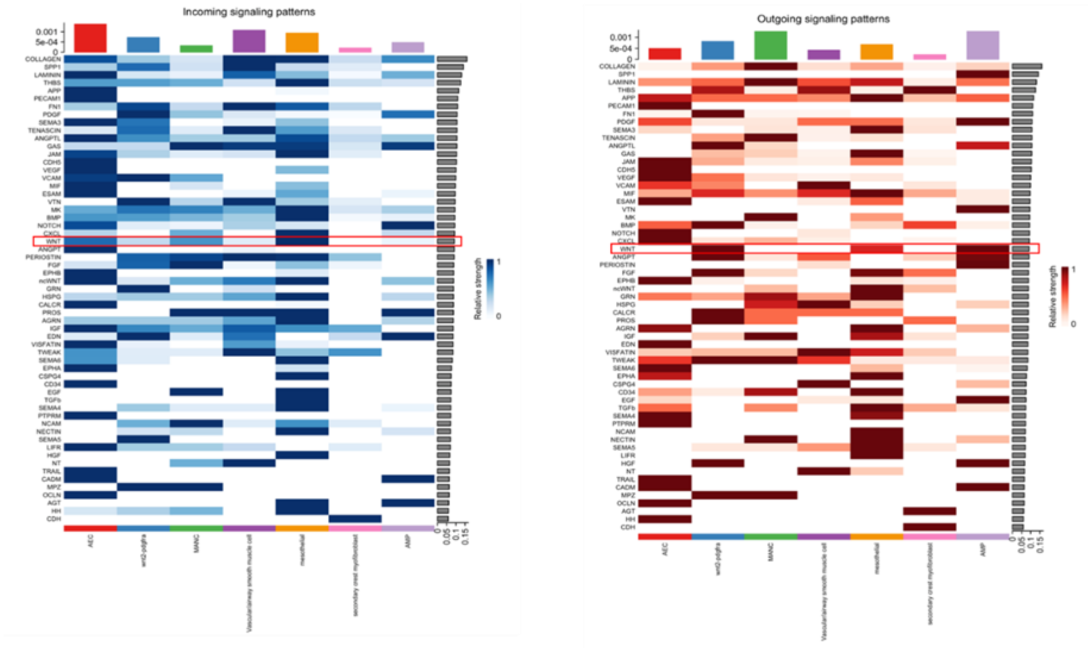

Supplemental Figure 3. Incoming and outgoing signals on CellChat. The intercellular network analysis was performed using the CellChat v1.6.1. The minimum cell count for filtering was set at 10 and only cell populations of interest were selected for the analysis.

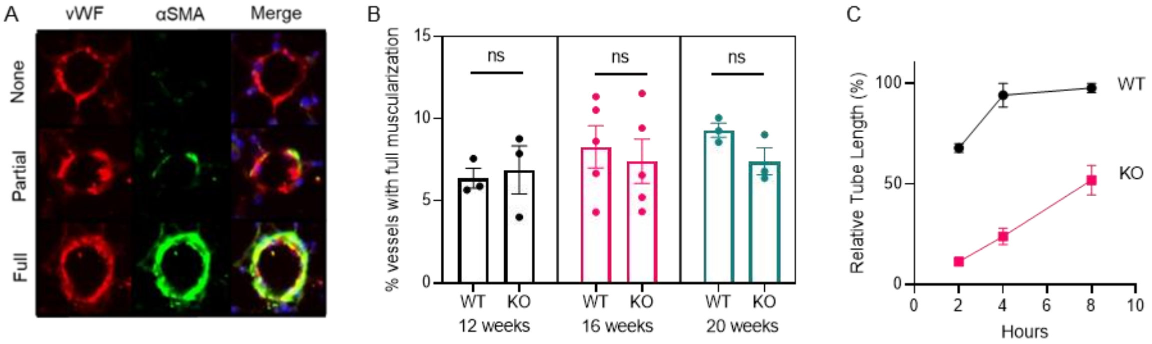

Supplemental Figure 4. Lung mesenchymal-specific mTORC1 activation did not alter vasculature count or remodeling at 8 weeks but significantly altered EC function. (A) Representative figures of scoring metric for vascular remodeling based on smooth muscle involvement. The degree of muscularization was defined by α-smooth muscle actin positive parts as percentage of the total pulmonary artery cross section: non-muscularized: < 20%, partial muscularization: 20-70%, fully muscularized: > 70%. (B) Vascular muscularization in 12-, 16- and 20-week old $Tbx4^{LME\_Cre}Tsc2^{WT}$ versus $Tbx4^{LME\_Cre}Tsc2^{KO}$ mice (n=3 in 12-week cohort, n=5 in 16-week cohort and n=3 in 20-week cohort). (C) Time to maximal tube formation in $Tsc2^{WT}$ (n=3) compared to $Tsc2^{KO}$ (n=3) mice.

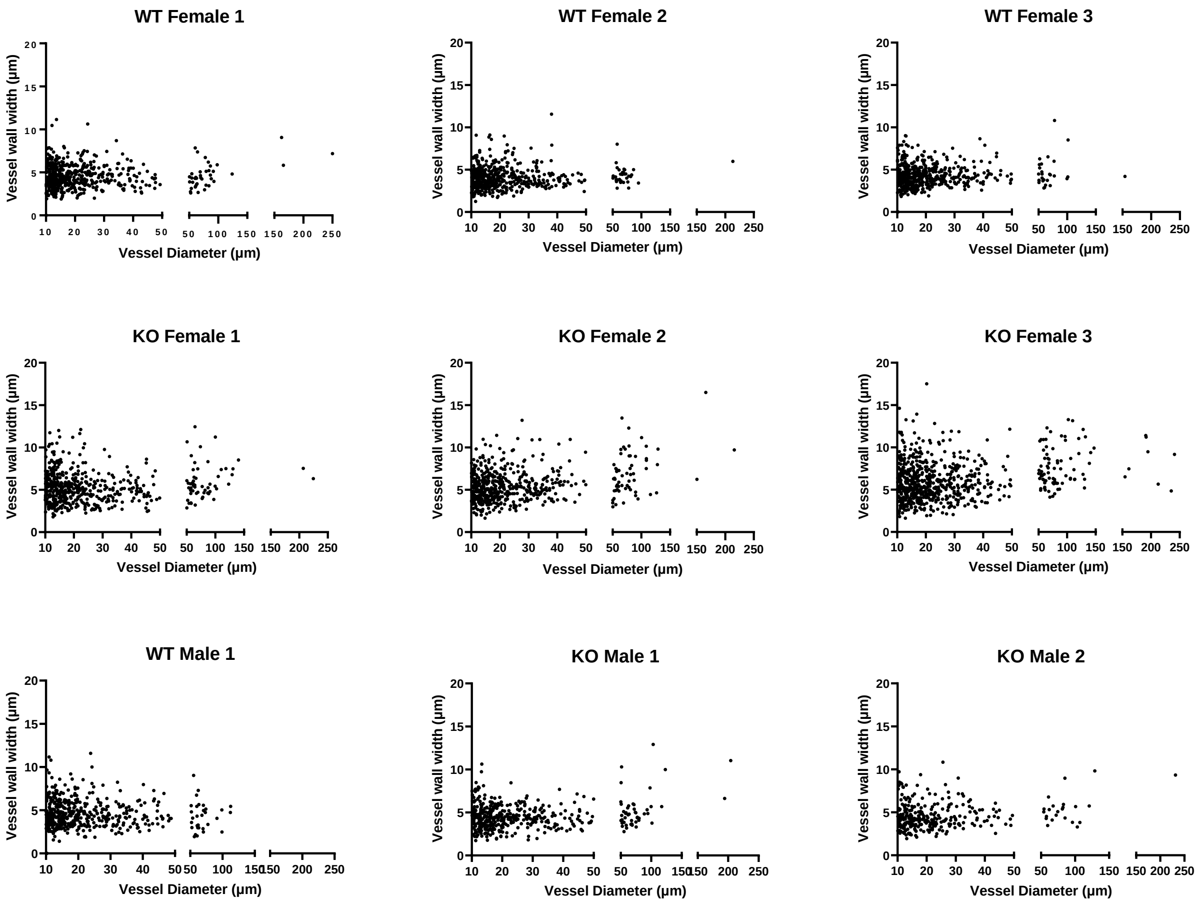

Supplemental Figure 5. Distribution of vessel wall thickness in individual mouse lungs. Vessel thickness was calculated as the distance between border of the vessel wall of the lumen to the vessel wall-lung tissue interface (encompassing the medial and intimal layer). $Tbx4^{LME\_Cre}Tsc2^{KO}$ mice had thicker vessels compared to age matched controls.

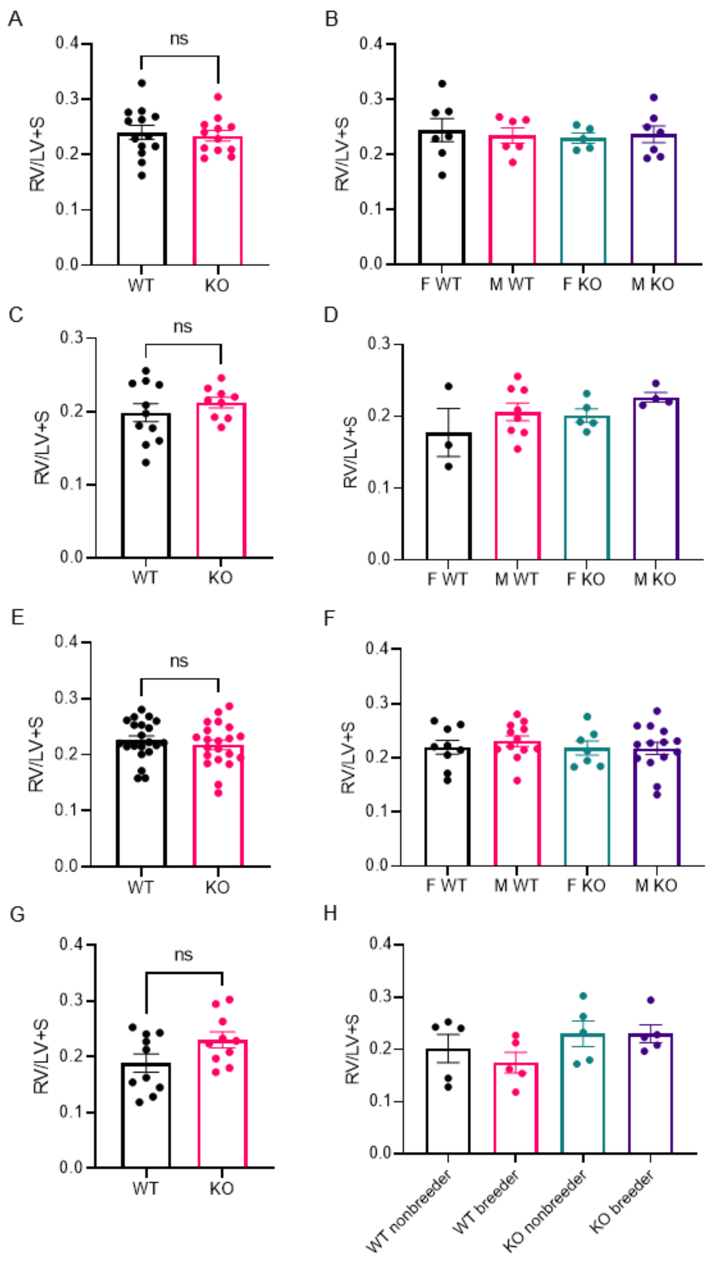

Supplemental Figure 6. Fulton Index in 12, 16, 20 and 24-week old *Tbx4*$^{LME\_Cre}$*Tsc2*^{WT} versus *Tbx4*$^{LME\_Cre}$*Tsc2*^{KO} mice. (A) 12-week-old mice, n= 13 WT, 12 KO. (B) 12-week-old mice by gender, n= 7 F WT, 5 M WT, 5 F KO, 7 M KO. (C) 16-week-old mice, n= 11 WT, 9 KO. (D) 16-week-old mice by gender, n=3 F WT, 8 M WT, 5 F KO, 4 M KO. (E) 20-week old mice, n= 21 WT, 21 KO. (F) 20-week-old mice by gender, n= 9 F WT, 12 M WT, 7 F KO, 14 M KO. (G) 24-week-old female mice, n = 10 WT, 10 KO. (H) 24-week-old mice by breeding status, n= 5 F WT, 5 M WT, 5 F KO, 5 M KO. Statistical analysis was performed using two-tailed Student's *t*-test or nonparametric Kruskal-Wallis ANOVA test.
