# Supplementary material for: Hyperactive mTORC1 in lung mesenchyme induces endothelial cell dysfunction and pulmonary vascular remodeling
Source: J Clin Invest. 2023 Dec 20;134(4):e172116. doi: 10.1172/JCI172116 (PMC10866655; doi:10.1172/JCI172116)
Supplement: Unedited blot and gel images [file jci-134-172116-s042.pdf]

Figure 5

Full Immunoblot – mouse ECs

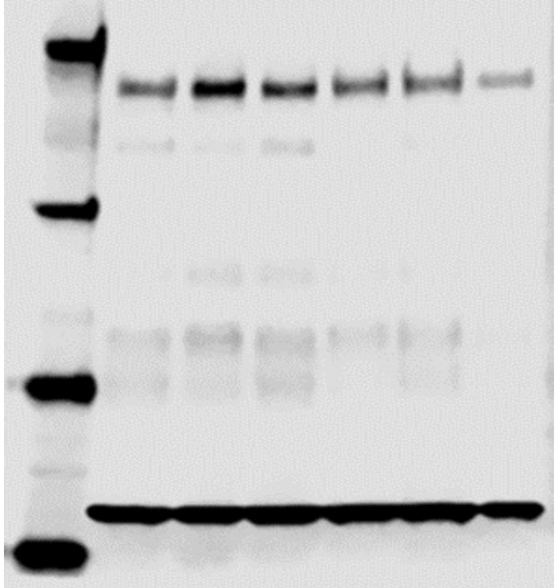

Endothelial Cells (8-week-old mice)

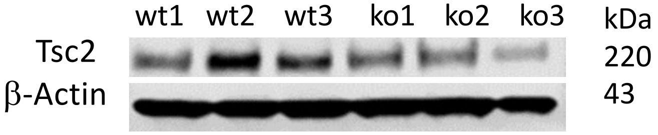


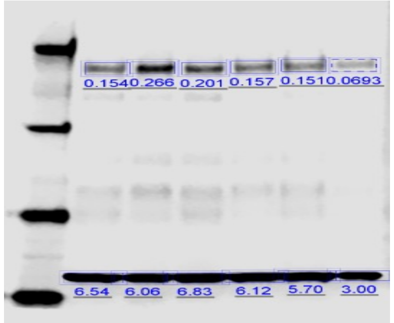


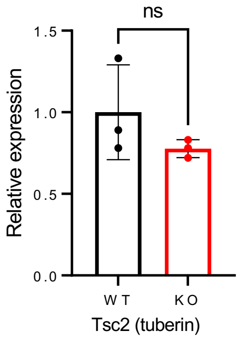


Prism Quantitation LiCor data: corrected for β-Actin in lane
Average control set as 1

| Table format: Grouped | Group A | | | Group B | | |
|---|---|---|---|---|---|---|
| | Wildtype | | | Tsc2ko | | |
| | A:1 | A:2 | A:3 | B:1 | B:2 | B:3 |
| 1 Tsc2 (tuberin) | 0.78 | 1.33 | 0.89 | 0.78 | 0.83 | 0.72 |

Figure 5

Full Immunoblot – mouse fibroblasts

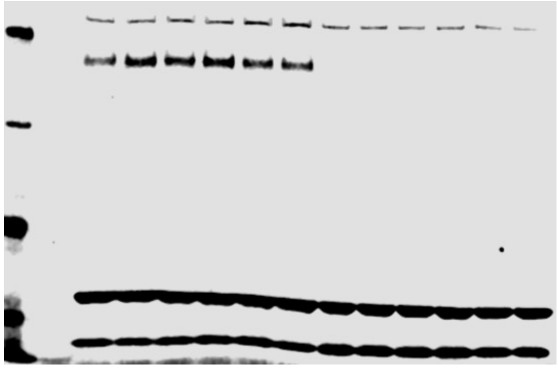


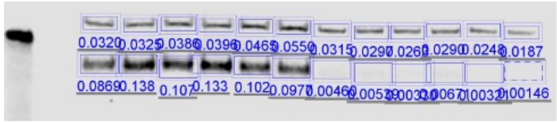

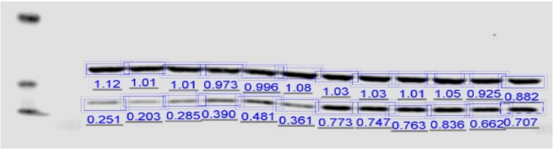

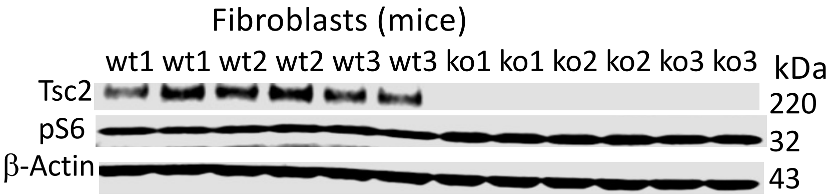


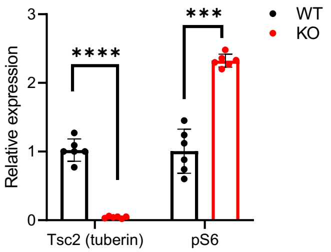


Prism Quantitation LiCor data: corrected for β-Actin in lane
Average control set as 1

| Table format: Grouped | Group A wildtype | | | | | | Group B Tsc2ko | | | | | |
|---|---|---|---|---|---|---|---|---|---|---|---|---|
| | A:1 | A:2 | A:3 | A:4 | A:5 | A:6 | B:1 | B:2 | B:3 | B:4 | B:5 | B:6 |
| 1 Tsc2 (tuberin) | 0.77 | 1.27 | 1.00 | 1.09 | 1.00 | 1.00 | 0.05 | 0.05 | 0.04 | 0.06 | 0.03 | 0.02 |
| 2 pS6 | 0.74 | 0.60 | 0.88 | 1.24 | 1.45 | 1.12 | 2.33 | 2.27 | 2.30 | 2.48 | 2.20 | 2.36 |

Supplementary 1

Full Immunoblot – human ECs

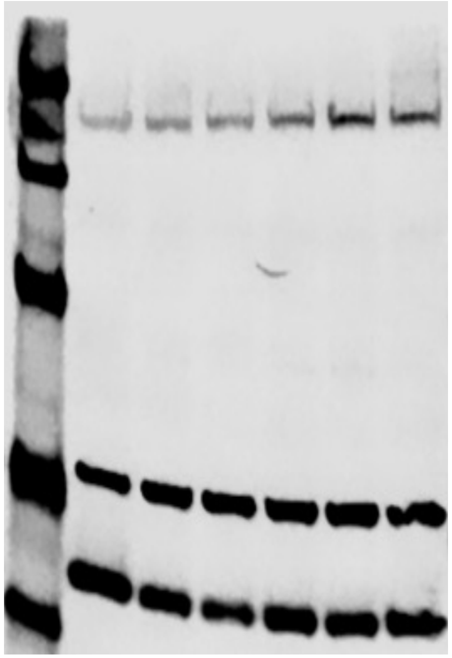

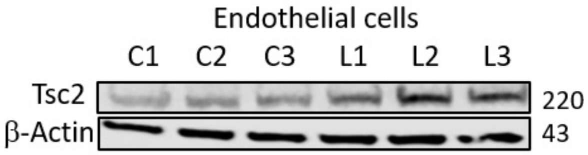


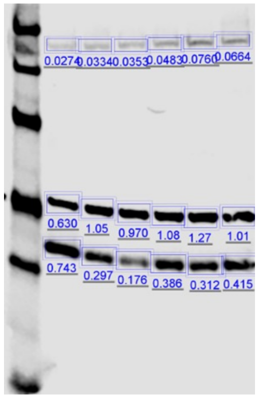


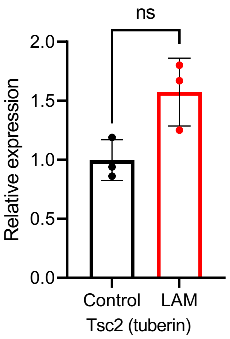


Prism Quantitation LiCor data: corrected for β-Actin in lane
Average control set as 1

| Table format: Grouped | Group A | | | Group B | | |
|---|---|---|---|---|---|---|
| | Control | | | LAM | | |
| | A:1 | A:2 | A:3 | B:1 | B:2 | B:3 |
| 1 Tsc2 (tuberin) | 1.19 | 0.86 | 0.94 | 1.25 | 1.67 | 1.80 |

Supplementary 1

Full Immunoblot – human fibroblasts

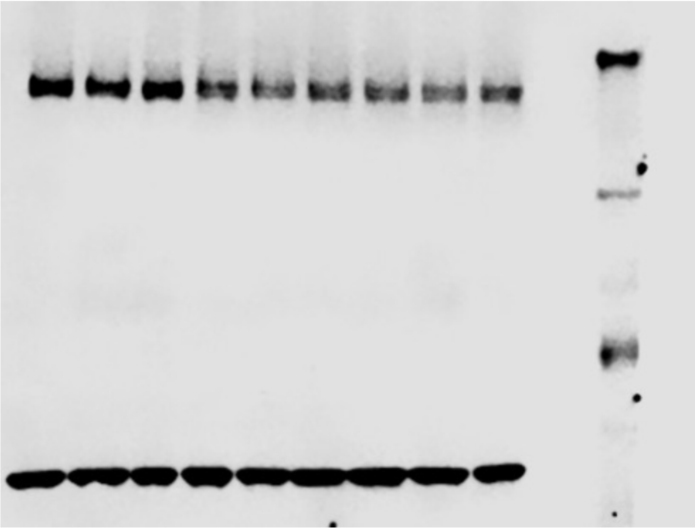

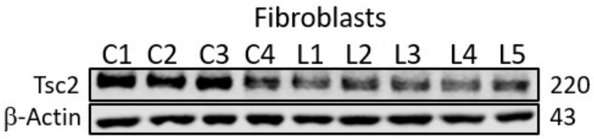


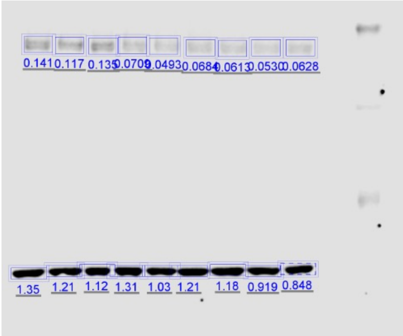


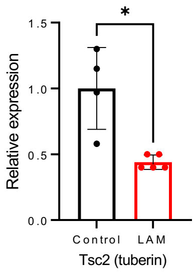


Prism Quantitation LiCor data: corrected for β-Actin in lane
Average control set as 1

| Table format: Grouped | | Group A Control | | | | | Group B LAM | | | | |
|---|---|---|---|---|---|---|---|---|---|---|---|
| | | A:1 | A:2 | A:3 | A:4 | A:5 | B:1 | B:2 | B:3 | B:4 | B:5 |
| 1 | Tsc2 (tuberin) | 1.15 | 0.97 | 1.30 | 0.58 | | 0.5 | 0.4 | 0.4 | 0.4 | 0.5 |
